# Discovery and functional prioritization of Parkinson’s disease candidate genes from large-scale whole exome sequencing

**DOI:** 10.1186/s13059-017-1147-9

**Published:** 2017-01-30

**Authors:** Iris E. Jansen, Hui Ye, Sasja Heetveld, Marie C. Lechler, Helen Michels, Renée I. Seinstra, Steven J. Lubbe, Valérie Drouet, Suzanne Lesage, Elisa Majounie, J. Raphael Gibbs, Mike A. Nalls, Mina Ryten, Juan A. Botia, Jana Vandrovcova, Javier Simon-Sanchez, Melissa Castillo-Lizardo, Patrizia Rizzu, Cornelis Blauwendraat, Amit K. Chouhan, Yarong Li, Puja Yogi, Najaf Amin, Cornelia M. van Duijn, International Parkinson’s Disease Genetics Consortium (IPGDC), Mike A. Nalls, Vincent Plagnol, Dena G. Hernandez, Manu Sharma, Una-Marie Sheerin, Mohamad Saad, Javier SimónSánchez, Claudia Schulte, Suzanne Lesage, Sigurlaug Sveinbjörnsdóttir, Sampath Arepalli, Roger Barker, Yoav Ben-Shlomo, Henk W. Berendse, Daniela Berg, Kailash Bhatia, Rob M. A. de Bie, Alessandro Biffi, Bas Bloem, Zoltan Bochdanovits, Michael Bonin, Jose M. Bras, Kathrin Brockmann, Janet Brooks, David J. Burn, Elisa Majounie, Steven Lubbe, Iris E. Jansen, Ryan Price, Aude Nicolas, Gavin Charlesworth, Codrin Lungu, Honglei Chen, Patrick F. Chinnery, Sean Chong, Carl E Clarke, Mark R Cookson, J. Mark Cooper, Jean Christophe Corvol, Carl Counsell, Philippe Damier, Jean-François Dartigues, Panos Deloukas, Günther Deuschl, David T. Dexter, Karin D. van Dijk, Allissa Dillman, Frank Durif, Alexandra Dürr, Sarah Edkins, Jonathan R. Evans, Thomas Foltynie, Jing Dong, Michelle Gardner, J. Raphael Gibbs, Alison Goate, Emma Gray, Rita Guerreiro, Clare Harris, Jacobus J. van Hilten, Albert Hofman, Albert Hollenbeck, Janice Holton, Michele Hu, Isabel Wurster, Walter Mätzler, Gavin Hudson, Sarah E. Hunt, Johanna Huttenlocher, Thomas Illig, Pálmi V. Jónsson, Jean-Charles Lambert, Cordelia Langford, Andrew Lees, Peter Lichtner, Patricia Limousin, Grisel Lopez, Delia Lorenz, Codrin Lungu, Alisdair McNeill, Catriona Moorby, Matthew Moore, Huw R. Morris, Karen E. Morrison, Valentina Escott-Price, Ese Mudanohwo, Sean S. O’Sullivan, Justin Pearson, Joel S. Perlmutter, Hjörvar Pétursson, Pierre Pollak, Bart Post, Simon Potter, Bernard Ravina, Tamas Revesz, Olaf Riess, Fernando Rivadeneira, Patrizia Rizzu, Mina Ryten, Stephen Sawcer, Anthony Schapira, Hans Scheffer, Karen Shaw, Ira Shoulson, Joshua Shulman, Ellen Sidransky, Colin Smith, Chris C. A. Spencer, Hreinn Stefánsson, Francesco Bettella, Joanna D. Stockton, Amy Strange, Kevin Talbot, Carlie M. Tanner, Avazeh Tashakkori-Ghanbaria, François Tison, Daniah Trabzuni, Bryan J. Traynor, André G. Uitterlinden, Daan Velseboer, Marie Vidailhet, Robert Walker, Bart van de Warrenburg, Mirdhu Wickremaratchi, Nigel Williams, Caroline H. Williams-Gray, Sophie Winder-Rhodes, Kári Stefánsson, Maria Martinez, Nicholas W. Wood, John Hardy, Peter Heutink, Alexis Brice, Thomas Gasser, Andrew B. Singleton, Huw R. Morris, Alexis Brice, Andrew B. Singleton, Della C. David, Ellen A. Nollen, Shushant Jain, Joshua M. Shulman, Peter Heutink

**Affiliations:** 1 German Center for Neurodegenerative Diseases (DZNE), Otfried-Müller-Str. 23, Tübingen, 72076 Germany; 2 Department of Clinical Genetics, VU University Medical Center, Amsterdam, 1081HZ The Netherlands; 3 Department of Molecular and Human Genetics, Baylor College of Medicine, Houston, TX USA; 4 Graduate School of Cellular & Molecular Neuroscience, Tübingen, 72074 Germany; 5 European Research Institute for the Biology of Aging, University of Groningen, University Medical Centre Groningen, Groningen, 9700AD The Netherlands; 6 Department of Clinical Neuroscience, UCL Institute of Neurology, London, UK; 7 Northwestern University Feinberg School of Medicine, Ken and Ruth Davee Department of Neurology, Chicago, IL USA; 8 Inserm U1127, CNRS UMR7225, Sorbonne Universités, UPMC Univ Paris 06, UMR_S1127, Institut du Cerveau et de la Moelle épinière, Paris, France; 9 Institute of Psychological Medicine and Clinical Neurosciences, MRC Centre for Neuropsychiatric Genetics and Genomics, Cardiff University, Cardiff, UK; 10 Laboratory of Neurogenetics, National Institute on Aging, Bethesda, MD USA; 11 Department of Molecular Neuroscience, UCL Institute of Neurology, London, UK; 12 Department of Medical & Molecular Genetics, King’s College London, London, UK; 13 Hertie Institute for Clinical Brain Research, University of Tübingen, Tübingen, Germany; 14 Department of Neurology, Baylor College of Medicine, Houston, TX USA; 15 Genetic Epidemiology Unit, Department of Epidemiology, Erasmus MC, Rotterdam, The Netherlands; 16 Assistance Publique Hôpitaux de Paris, Hôpital de la Salpêtrière, Département de Génétique et Cytogénétique, Paris, France; 17 Department of Neuroscience and Program in Developmental Biology, Baylor College of Medicine, Houston, TX USA; 18 Jan and Dan Duncan Neurological Research Institute, Texas Children’s Hospital, 1250 Moursund St., N.1150, Houston, TX 77030 USA

**Keywords:** Parkinson’s disease, Genomics, Whole-exome sequencing, Loss-of-function, Rare variants, Functional screening, Mitochondria, Parkin, α-synuclein, Animal model

## Abstract

**Background:**

Whole-exome sequencing (WES) has been successful in identifying genes that cause familial Parkinson’s disease (PD). However, until now this approach has not been deployed to study large cohorts of unrelated participants. To discover rare PD susceptibility variants, we performed WES in 1148 unrelated cases and 503 control participants. Candidate genes were subsequently validated for functions relevant to PD based on parallel RNA-interference (RNAi) screens in human cell culture and *Drosophila* and *C. elegans* models.

**Results:**

Assuming autosomal recessive inheritance, we identify 27 genes that have homozygous or compound heterozygous loss-of-function variants in PD cases. Definitive replication and confirmation of these findings were hindered by potential heterogeneity and by the rarity of the implicated alleles. We therefore looked for potential genetic interactions with established PD mechanisms. Following RNAi-mediated knockdown, 15 of the genes modulated mitochondrial dynamics in human neuronal cultures and four candidates enhanced α-synuclein-induced neurodegeneration in *Drosophila*. Based on complementary analyses in independent human datasets, five functionally validated genes—*GPATCH2L*, *UHRF1BP1L*, *PTPRH*, *ARSB*, and *VPS13C*—also showed evidence consistent with genetic replication.

**Conclusions:**

By integrating human genetic and functional evidence, we identify several PD susceptibility gene candidates for further investigation. Our approach highlights a powerful experimental strategy with broad applicability for future studies of disorders with complex genetic etiologies.

**Electronic supplementary material:**

The online version of this article (doi:10.1186/s13059-017-1147-9) contains supplementary material, which is available to authorized users.

## Background

Next-generation sequencing (NGS) approaches have recently accelerated the identification of variants responsible for familial Parkinson’s disease (PD) [1–4]. While a positive family history is common in PD, large, multigenerational pedigrees, especially with available DNA and clinical evaluations, remain exceptional, hindering progress in unraveling the genetic underpinnings. Importantly, several genes initially discovered to cause PD in families, such as *LRRK2*, *GBA*, and *PARK2/parkin*, were subsequently discovered with surprisingly high frequency in “sporadic” PD cohorts [5, 6]. To date, large population samples of individuals with PD have primarily contributed to the discovery of common variant susceptibility loci, based on genome-wide association studies (GWAS) of case/control cohorts [7]. The variants identified by GWAS have modest effect sizes and collectively fail to account for current estimates of PD heritability [8, 9]. Considering the above, it seems likely that additional less common alleles, with larger effect sizes, contribute to PD risk in the population and NGS is one promising approach to identify such alleles. Despite recent successes in other neurodegenerative disease with complex genetic etiologies, including Alzheimer’s disease [10–12] and amyotrophic lateral sclerosis [13, 14], sequencing has yet to be deployed in large, unrelated PD case/control samples for rare variant discovery.

The successful discovery of rare variant risk alleles in population-based PD samples faces a number of potential challenges. Perhaps most importantly, analyses of rare variants in large family pedigrees is greatly facilitated by segregation analysis which is not possible in cohorts of unrelated individuals, leading to an increased number of candidate variants to consider. Assumptions of a recessive inheritance model and the application of stringent filters, such as consideration of only strongly damaging, loss-of-function (LoF) variants, is one potential solution, but this is likely to miss many important variants, including dominantly acting alleles. Further, PD is characterized by extensive genic and allelic heterogeneity and extremely large cohorts may be required to document sufficient numbers of cases to facilitate meaningful statistical comparisons [15]. Lastly, as PD is: (1) common (~1–3% prevalence); (2) strongly age-dependent; and (3) often preceded by a prolonged presymptomatic or minimally symptomatic phase, we may expect to find truly pathogenic rare variants, including those with large effect sizes, in “control” cohorts of adults (due to unrecognized or early disease stages with minimal symptoms). Therefore, given the occurrence of rare variants, including potentially damaging variants, in most genomes of presumably healthy individuals [16], it may be difficult to identify genes/variants that truly cause disease. Importantly, recent advances in cellular and animal models, along with improved understanding of PD pathogenesis, enable an integrated approach, in which variant discovery is coupled with a functional screening pipeline for prioritization of those genes worthy of more intensive study.

In this collaborative study of the International Parkinson’s Disease Genomics Consortium (IPDGC), we report the results of whole-exome sequencing (WES) in 1148 PD cases, the largest such cohort examined to date. Consistent with the younger age of PD onset in this cohort, which is often associated with a recessive inheritance [17–19], and to prioritize candidate genes/variants for initial investigation, our analysis focuses on genes with homozygous or compound heterozygous LoF variants. We further couple the human genetic studies with functional screening in mammalian cell culture and invertebrate animal models, successfully identifying those candidate genes showing interactions with established PD mechanisms, including mitochondrial dynamics and α-synuclein-mediated neurodegeneration. Although no sufficiently powered exome dataset was available for definitive replication, human genetic validation was undertaken in several independent datasets. Our integrated approach identifies five strong candidate PD susceptibility genes worthy of further investigation, and exemplifies a powerful strategy with potential broad applicability to the follow-up of future rare variant studies in PD and other neurologic disorders with complex genetic etiologies.

## Results

### Discovery of recessive LoF variants from PD exomes

A total of 920,896 variants (93.2% single nucleotide variants and 6.8% insertions and deletions) were called in a WES dataset of 1651 participants, including 1148 young-onset PD cases (average age of onset, 40.6 years; range, 5–56 years) and 503 control participants with European ancestry. As our cohort has an average age at onset of less than 45 years, we focused our search on homozygous and putative compound heterozygous variants, consistent with a recessive inheritance model. Although most PD cases were prescreened for mutations in established PD genes, we identified two participants with homozygous exonic variants in *parkin* and *PINK1* (Additional file 1: Table S1). In order to identify novel PD gene candidates, we focused on variants that are rare in control populations. Considering the worldwide prevalence for PD (0.041% in individuals aged 40–49 years) [20], we used a minor allele frequency (MAF) threshold of 1% and only considered LoF variants causing a premature stop codon or splicing site mutations (see “Methods”). When co-occurring with a heterozygous LoF variant, we also considered rare, heterozygous amino-acid changing missense alleles that were predicted to be deleterious (CADD > 20), consistent with a compound heterozygous recessive genotype.

**Fig. 1:**
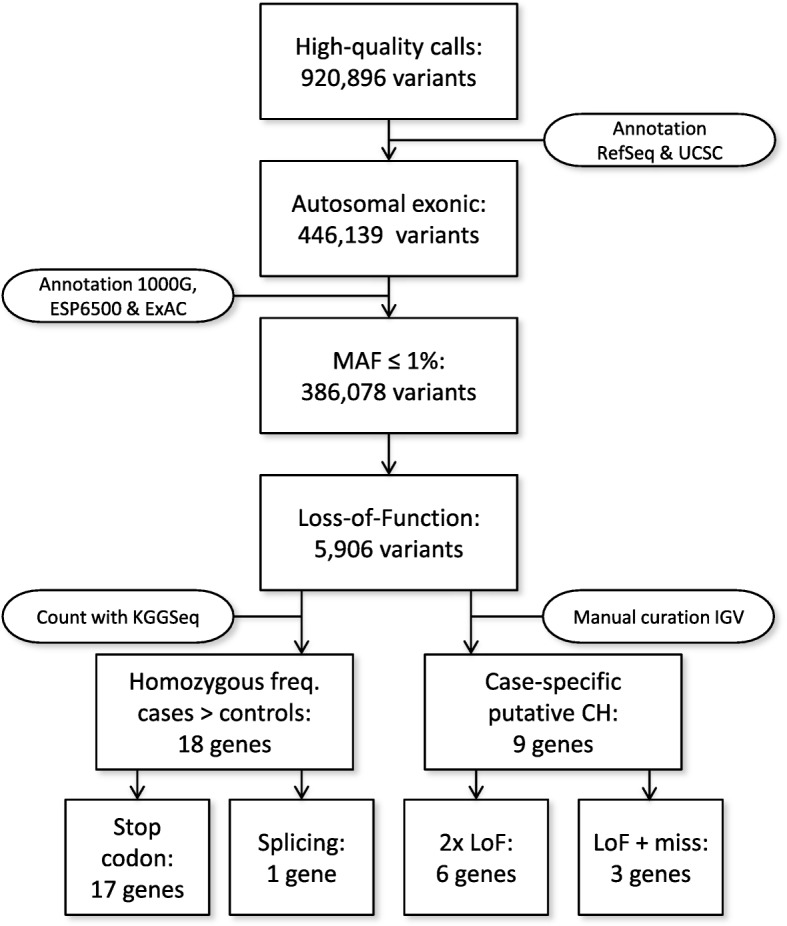
*Flowchart* explaining multiple filtering steps to select LoF variants with assumed recessive inheritance pattern. Functional annotation was performed with transcripts of RefSeq and UCSC databases. MAF annotations were based on 1000 Genomes project, Exome variant Server, and the ExAC database. Seventeen genes harbored homozygous variants causing stopgain or loss and one gene contained a homozygous splicing variant. For the putative compound heterozygous genes, six genes were selected based on the presence of two LoF variants, and three genes were based on the presence of one LoF variant and one missense variant (predicted to belong to the 1% most harmful variants of the genome)

Figure 1 displays each variant filtering step along with the corresponding numbers of implicated variants. Following Sanger sequencing confirmation, we identified a total of 27 candidate genes—18 genes encompassing homozygous variants and nine genes harboring putative compound heterozygous variants—all predicted to cause a loss of gene function (Table 1). Approximately 17% of the variants are absent in public allele frequency databases (1000 Genomes Project (1000G), Exome Sequencing Project v. 6500 (ESP6500), or Exome Aggregation Consortium (ExAC)) and therefore implicated to be novel. Except in the case of *ARSB*, the other 26 genes harbor LoF variants in only a single case, consistent with the hypothesis that novel recessive PD alleles may consist of many rare, “private” mutations. Four PD cases in our cohort were identified with a LoF variant in the *ARSB* gene, in which mutations have previously been linked with the recessive lysosomal storage disorder, MPS VI (also called Maroteaux-Lamy syndrome). All four individual cases, along with one control participant, were homozygous for a variant (rs138279020) predicted to disrupt splicing. Although this variant is neither reported in ExAC nor was frequency information available from dbSNP, the MAF was 0.065 in our cohort (MAF_CASES_ = 0.073, MAF_CONTROLS_ = 0.052, *p* = 0.054). Although relatively frequent in our control dataset (MAF > 1%), we have retained it among our results, based on three considerations. First, information was not present in dbSNP, ExAC, or ESP6500, which was the basis for applying this frequency filter in all other cases. Second, at least one of the homozygous individuals had clinical manifestations consistent with MPS VI, supporting potential pathogenicity of this allele (see “Discussion”). Lastly, as detailed below, our functional studies identify links between manipulation of ARSB and cellular/organismal phenotypes consistent with a potential role in PD.

**Table 1:** Recessive LoF variants

WES results		Variant characteristics					Functional information				Validation	
					ExAC (EU)							
Gene	Type	Chr:bp (hg19)	Ref/Alt all	dbSNP137	MAF	*n* HZ (freq.)	Variant type	AA change	AA length	CADD	Functional	Genetic
*ANKRD30A*	CH	10:37438759	G/T	x	x	x	stopgain	NM_052997:p.E487X	1341	35.0		
		10:37508648	A/AT	x	0.0031%	0	fs insertion	NM_052997:p.Q1280fs	1341	24.2		
*ARSB*	HZ	5:78281383	T/TA	rs138279020	x	x	splicing	NM_198709:c.190-2insT	533	2.2	S	neuroX
*c11orf21*	HZ	11:2321829	C/T	rs74048215	0.037%	0	stopgain	NM_001142946:p.W69X	178	35.0		
*CALML4*	HZ	15:68497597	G/A	rs11071990	0.75%	1 (0.00003%)	stopgain	NM_001031733:p.R40X	120	25.9		
*CAPS2*	HZ	12:75687045	C/CTATTTGAT	x	0.68%	4 (0.00012%)	stopgain	NM_001286547:p.L321X	382	33.0		
*CD36*	CH	7:80285955	C/T	x	0.0075%	0	stopgain	NM_001127444:p.Q74X	472	26.5		GRIP
		7:80302705	C/T	x	0.011%	0	missense	NM_001127444:p.P412S	472	25.9		
*COL6A5*	CH	3:130132401	C/T	rs190283135	0.12%	0	stopgain	NM_001278298:p.Q1559X	2611	38.0		
		3:130107827	G/A	x	0.32%	0	missense	NM_001278298:p.V756M	2611	23.0		
*DIS3*	CH	13:73355096	G/A	rs201493043	0.0045%	0	stopgain	NM_014953:p.Q92X	958	37.0	Mm	
		13:73333935	A/G	rs141067458	0.22%	0	stoploss	NM_014953:p.X959Q	958	14.1		
*FAM71A*	HZ	1:212799882	A/T	rs143861665	0.80%	2 (0.00006%)	stopgain	NM_153606:p.K555X	594	36.0	Mm	
*FAM83A*	CH	8:124195352	G/T	rs148011353	0.23%	2 (0.00006%)	stopgain	NM_032899:p.G86X	434	26.2		PPMI
		8:124195506	T/G	x	x	x	missense	NM_032899:p.V137G	434	25.6		
*GH2*	HZ	17:61957946	C/T	rs150668018	0.33%	1 (0.00003%)	stopgain	NM_022557:p.W214X	256	26.8		
*GPATCH2L*	HZ	14:76644266	C/T	rs117516637	0.53%	1 (0.00003%)	stopgain	NM_017972:p.R362X	482	18.8	Mp,Mm	PPMI
*KALRN*	HZ	3:124303696	C/T	rs56407180	0.39%	0	stopgain	NM_007064:p.R10X	1289	37.0	Mp	
*KCNK16*	HZ	6:39282816	G/A	rs138573996	0.44%	2 (0.00006%)	stopgain	NM_001135107:p.Q251X	322	9.5	Mm	
*MNS1*	CH	15:56736045	G/A	x	x	x	stopgain	NM_018365:p.Q232X	495	38.0	Mm	
		15:56748680	G/A	x	0.003%	0	stopgain	NM_018365:c.C265T:p.Q89X	495	37.0		
*OR7G3*	HZ	19:9236969	G/A	rs61751875	0.15%	1 (0.00003%)	stopgain	NM_001001958:p.R220X	312	35.0	Mm	
*PCDHA9*	HZ	5:140229907	C/G	x	0.0046%	0	stopgain	NM_014005:p.Y609X	950	34.0	Mm	
*PTCHD3*	HZ	10:27688101	G/A	rs142646098	0.97%	6 (0.00018%)	stopgain	NM_001034842:p.R476X	767	34.0	Mp,Mm	
*PTPRH*	CH	19:55697712	G/A	rs147881000	0.39%	1 (0.00003%)	stopgain	NM_002842:p.Q887X	1115	36.0	S,Mm	neuroX
		19:55716715	C/A	rs201517965	0.0030%	0	stopgain	NM_002842:p.E200X	1115	26.0		
*PZP*	CH	12:9321534	G/A	rs145240281	0.85%	4 (0.00012%)	stopgain	NM_002864:p.R680X	1482	32.0		
		12:9333626	G/A	rs117889746	0.021%	0	stopgain	NM_002864:p.Q598X	1482	36.0		
*SSPO*	HZ	7:149493519	C/T	rs57595625	0.011%	0	stopgain	NM_198455:p.Q2199X	5150	10.4		
*SVOPL*	HZ	7:138341219	G/A	rs117871806	0.35%	0	stopgain	NM_174959:p.R18X	492	38.0	Mp,Mm	
*TCHHL1*	HZ	1:152058192	G/A	rs150014958	0.037%	0	stopgain	NM_001008536:p.Q656X	904	35.0	Mp	
*TMEM134*	HZ	11:67235051	G/A	rs143199541	0.70%	3 (0.00009%)	stopgain	NM_001078650:p.R84X	195	34.0	S	
*UHRF1BP1L*	HZ	12:100433523	T/A	x	x	x	stopgain	NM_015054:p.K1376X	1464	35.0	Mm	neuroX
*VPS13C*	CH	15:62174851	C/A	x	x	x	stopgain	NM_017684:p.E3147X	3628	55.0	S,Mm	neuroX,GWAS
		15:62305257	C/CTCTG	x	x	x	fs insertion	NM_017684:p.R226fs	3628	27.4		
*ZNF543*	HZ	19:57838058	G/A	rs150392165	0.60%	1 (0.00003%)	stopgain	NM_213598:p.W68X	600	25.0	Mp,Mm	

The current designation of rs138279020 is rs11424557

*Type* type of affecting 2 alleles, *CH* putative compound heterozygote, *HZ* homozygote, *Chr:bp* chromosome and base-pair position, *hg19* reference build, *Ref* reference, *Alt* alternative, *ExAC* Exome Aggregation consortium, *EU* European, *fs* frameshift, *AA change* amino acid change for specified RefSeq transcript, *MAF* minor allele frequency, *n HZ* number of individuals with homozygous variant, *AA length* length of specified transcript in amino acids, *PPMI* Parkinson Progression Markers Initiative, *CADD* functional algorithm prediction (>20 = belongs to 1% most damaging variants of total genome), *Mp* mitochondrial Parkin translocation assay, *Mm* mitochondrial morphology assay, *S* α-synuclein assay

Of note, while the analyses of the IPDGC WES dataset and subsequent work described here were in progress, an independent family-based sequencing study identified *VPS13C* as a cause of autosomal recessive parkinsonism [21]. Although the single IPDGC subject with compound heterozygous *VPS13C* LoF alleles was published as a replicate case in that work, we retained it among the 27 candidates described here, since it was independently carried forward for all analyses detailed below.

### Tolerability of gene LoF in humans and animal models

The “tolerability” of recessive LoF genotypes has important implications for understanding the genetic basis of adult-onset, age-influenced disorders such as PD. As most of the identified homozygous and putative compound heterozygous LoF genotypes are based on a single individual, we also examined for their occurrence in a large, recently published study [16] of predicted complete gene knock-outs in the Icelandic population, including 104,220 participants with imputed genotypes, based on whole genome sequencing from a subset of 2363 individuals. The Icelandic population is enriched for rare disease-causing mutations with a recessive inheritance pattern, given a strong founder effect and non-random mating patterns. Twelve of the variants that we identified are also present in the Icelandic study (Additional file 1: Table S2); however, the observed homozygote frequencies are not sufficiently high to confidently exclude them as possible PD genes and importantly, detailed phenotypic data are not publicly available for these participants. For example, 29 Icelandic participants are reported homozygous for the identical *PTCHD3* stopgain variant (c.C1426T, p.R476X) as the single PD case in our WES study. However, this is only 0.028% of the total sample set and below the reported prevalence of young-onset PD (0.041%).

We additionally examined for the presence of other LoF variants with a recessive inheritance pattern in our implicated candidate genes (Additional file 1: Table S2). For a subset of genes, we indeed identified several variants with particularly high homozygote frequencies including *OR7G3* (9.16%), *SSPO* (9.38%), and *PTCHD3* (16.55%). This is consistent with prior reports describing a homozygous deletion covering *PTCHD3* in apparently healthy individuals, consistent with a non-essential role [22]. Assuming that the variants in *OR7G3*, *SSPO*, and *PTCHD3* confer similar LoF to the alleles identified in our PD WES data, their high variant frequency makes these genes unlikely to be highly penetrant PD-risk loci.

Human genes harboring homozygous LoF variants—especially those observed recurrently in large population-based datasets—potentially identify genes that are dispensable for fetal and subsequent child development. Given the limited human phenotypic information available, we further investigated the potential tolerability for the implicated genes using a cross-species approach, performing systematic LoF analysis in the nematode, *C. elegans*. Out of the 27 candidate genes identified in our WES analysis, ten were well conserved in the *C. elegans* genome and nine had readily available RNA-interference (RNAi) reagents for LoF screening (see “Methods”). Each gene was targeted for knockdown using RNAi and we assessed for developmental lethality and survival. The results of these studies, along with other LoF data from public databases, are available in Additional file 1: Table S3. Knockdown of homologs of *DIS3* (*dis-3*), *KALRN* (*unc-73*), and *PTCHD3* (*ptr-10*) resulted in developmental arrest and/or reduced survival in *C. elegans*. Notably, homologs of *KALRN* and *DIS3* are also associated with reduced viability following genetic disruption in both *Drosophila* [23, 24] and mice [25, 26]. Thus, these results are potentially consistent with conserved, early, and/or essential developmental roles for these genes and the absence of individuals harboring homozygous LoF variants in the Icelandic cohort [16].

Since the human genome contains multiple gene paralogs for *KALRN* and *PTCHD3*, genetic redundancy might account for how LoF might be tolerated in humans but not in simple animal models. Alternatively, it is possible that the allelic variants implicated in our PD WES cohort and Icelandic study might not cause a complete LoF (i.e. genetic null) despite the algorithmic predictions, instead causing only a partial LoF. Nevertheless, these cross-species comparisons suggest essential and early developmental roles for homologs of *PTCHD3*, *DIS3*, and *KALRN*, and informing our consideration of potential contribution to adult-onset disorders, such as PD.

### Variant aggregation analyses

For the 27 genes implicated based on our primary analyses of homozygous or compound heterozygous LoF variants, we additionally considered evidence for the presence of other allelic variants conferring risk for PD in our cohort. We therefore performed burden analyses leveraging our IPDGC WES data, testing two nested classes of variants: (1) a subset predicted to be deleterious (CADD > 20); and (2) all amino-acid changing missense alleles. Rare variants (MAF < 0.018) were considered either selectively or in joint models with common variants (MAF > 0.018). As detailed in Additional file 1: Table S4, the rare variant aggregation association analyses provided further evidence in support of four candidate genes: *GH2*, *PTPRH*, *UHRF1BP1L*, and *ZNF453*. Interestingly, the burden association at the *PTPRH* gene is further enhanced when common and rare variants are simultaneously modeled.

Our analyses of LoF variants in PD exomes identify a number of promising candidate genes. However, even though a positive family history was observed for almost 40% of the cases, segregation analysis of the variants in families is not feasible, as DNA samples are not available from additional family members. Further, since most of the genes implicated contribute to single or few cases, we are unable to perform meaningful statistical comparisons, based on the limited numbers of LoF variants identified by WES in cases versus controls. As an alternative strategy, we therefore deployed a combination of cell-based and model organism functional screens to define potential links between the 27 candidate genes (Table 1) and well-established mechanisms of PD susceptibility and pathogenesis, including (1) mitochondrial health and (2) *α-*synuclein-mediated toxicity.

### Functional prioritization: mitochondrial health

Although the mechanism of neurodegeneration in PD remains incompletely defined and may be heterogeneous, mitochondrial dysfunction has been proposed to play an important role, particularly in young onset PD [27–29]. Notably, *parkin (PARK2)*, *DJ-1*, and *PINK1*, associated with autosomal recessive, juvenile-onset Parkinsonism, have roles in mitochondrial dynamics and quality control [30]. Specifically, Parkin is an E3 ubiquitin ligase and recruited selectively to dysfunctional mitochondria with a low membrane potential [31]. Further, the neurotoxicity of α-synuclein, the primary constituent of Lewy body inclusions in PD, has also been linked to mitochondrial injury [32]. We therefore hypothesized that LoF in candidate genes identified from our analyses of WES, might similarly impact mitochondria, consistent with roles in PD susceptibility.

**Fig. 2:**
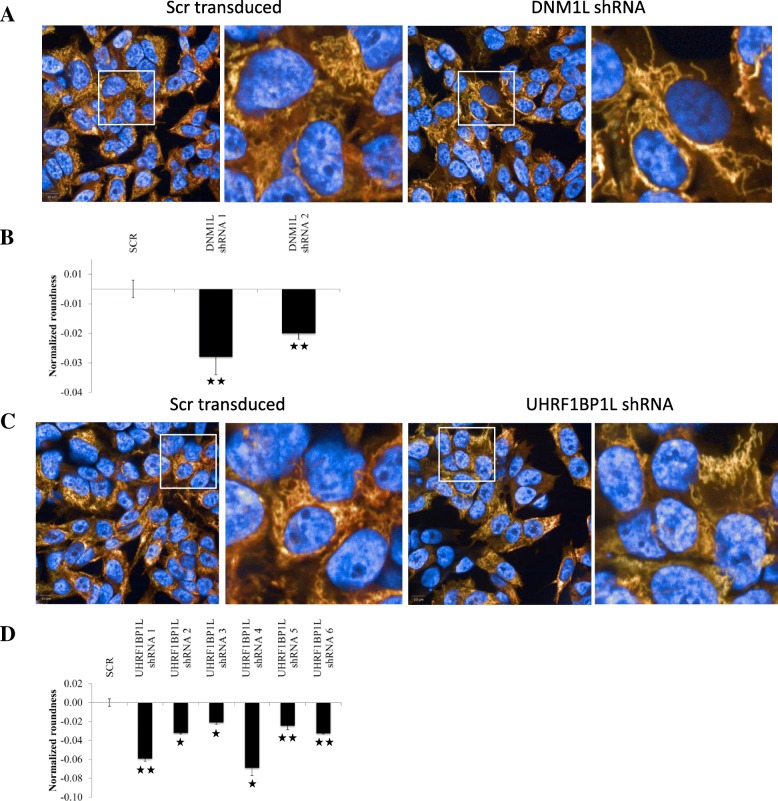
High-content assay for mitochondrial morphology. Effect of *DNM1L* shRNA (**a**, **b**) and *UHRF1BP1L* shRNA (**c**, **d**). BE(2)M17 cells stained with Hoechst (*blue*; nuclei), MitoTracker CMXros, and MitoTracker Deepred (*yellow*; mitochondria). **a** Cells infected with shRNA encoding a scrambled sequence (SCR, *left panel*) and decrease in mitochondrial axial length ratio and roundness for *DNM1L* (positive control, *right panel*). **b** The graph displays normalized mitochondrial roundness. **c** Cells infected with shRNA encoding a SCR sequence (*left panel*) and decrease in number of mitochondria per cell, mitochondrial axial length ratio, and roundness for *UHRF1BP1L* (*right panel*). **d** The graph displays normalized mitochondrial roundness. Data are median values ± median absolute deviation (MAD) of N = 6 measurements. *p < 0.05 and **p < 0.01, Mann–Whitney *U* test (see “Methods”). All values were normalized to the negative control (infected with SCR shRNA) and all shRNA clones that meet the cutoff criteria are shown (**b**, **d**)

Therefore we quantified mitochondrial morphology after gene knockdown in BE(2)-M17 neuroblastoma cells by examining three parameters commonly used for quantification of mitochondrial morphology: mitochondrial number, axial length ratio, and roundness [33]. Cells transduced with the short hairpin RNA (shRNA) encoding a scrambled sequence were used for normalization and positive controls for mitochondrial morphology were included in each experiment. For example, knockdown of the mitochondrial fission gene dynamin 1-like (*DNM1L*), a positive control, results in elongated mitochondria and therefore decreases mitochondrial axial length ratio and roundness (Fig. 2a, b) [34]. Knockdown of 13 genes show a significant effect on at least one of the three parameters (Additional file 1: Table S5 and Table S6 and Additional file 2: Figure S1). *GPATCH2L* shows the largest increase in mitochondrial roundness, while *UHRF1BP1L* displays the largest decrease (Fig. 2c, d).

**Fig. 3:**
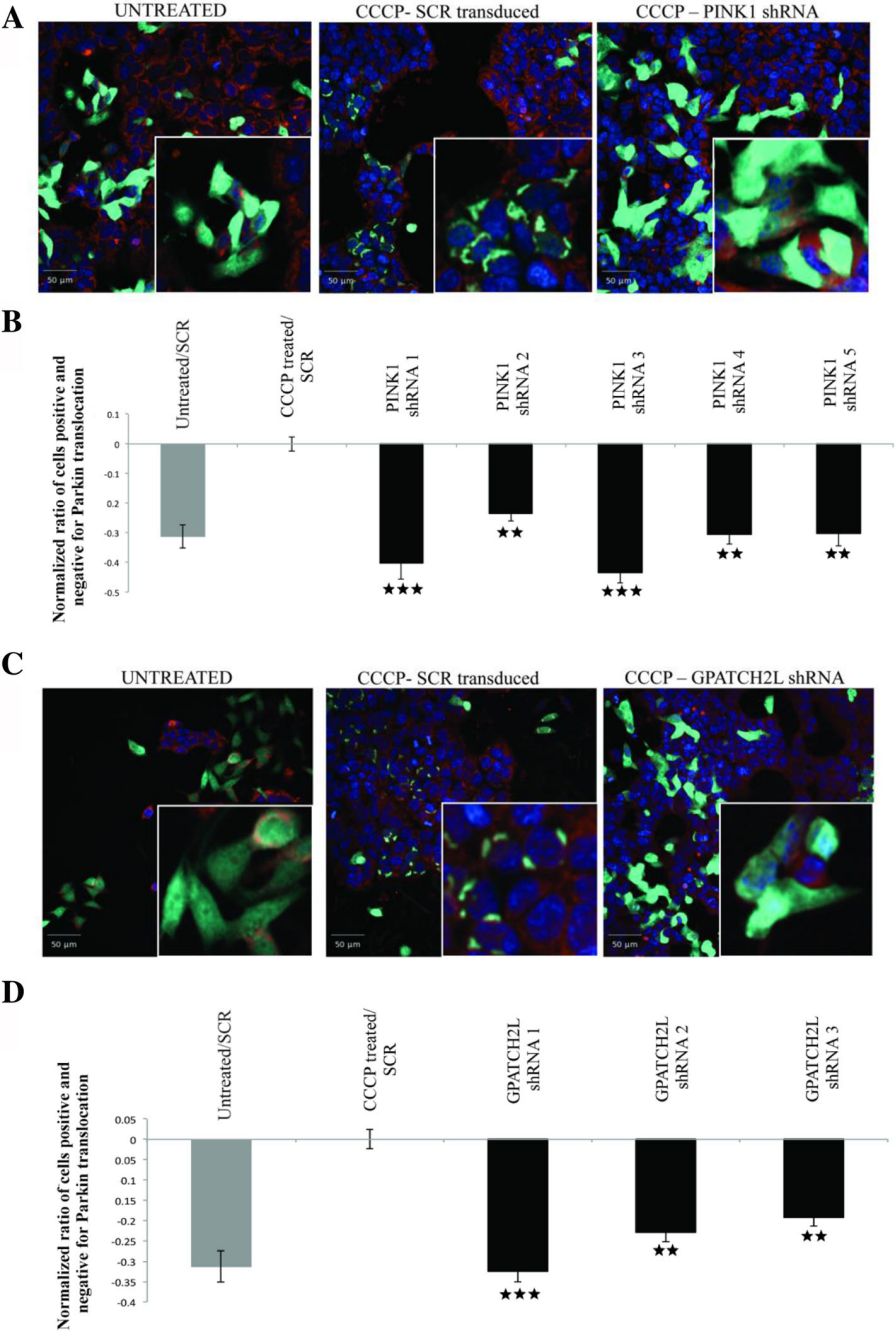
High content assay for Parkin translocation. Effect of *PINK1* shRNA (**a**, **b**) and *GPATCH2L* shRNA (**c**, **d**). **a**, **c** Cells are labeled for nuclei (*blue*; Hoechst), Parkin-GFP (*green*), mitochondria (*red*, Mitotracker Deepred). Untreated cells infected with shRNA encoding a scrambled sequence show absence of puncta (*left panel*). Cells infected with a scrambled sequence but treated with CCCP show a significant increase in puncta formation (*middle panel*). Infection of cells with shRNA targeting *PINK1* or *GPATCH2L* prevents the accumulation of Parkin on mitochondrial (*right panel*). **b**, **d** The *graph* displays the normalized ratio of cells positive for translocation and cells negative for parkin translocation. All values were normalized to the negative control (CCCP treated infected with shRNA encoding a scrambled sequence). Data are median values ± median absolute deviation (MAD) of N = 6 measurements. *p < 0.05, **p < 0.01, and ***p < 0.001, Mann–Whitney *U* test (see “Methods”). All shRNA clones that meet the cutoff criteria (see “Methods”) are shown

We also took advantage of a well-established Parkin translocation assay [31, 35–38] based on BE(2)-M17 human neuroblastoma cells stably expressing Parkin-GFP. As expected, upon exposure to the mitochondrial toxin and electron transport chain uncoupling reagent, CCCP, we observed robust translocation of Parkin-GFP from the cytoplasm (Fig. 3a, untreated) to the mitochondria (Fig. 3a, CCCP-SCR transduced) and this was PINK1-dependent (Fig. 3a, CCCP-PINK1 shRNA), which provides an internal, positive control in our assay. CCCP-induced Parkin accumulation was assessed by high-content microscopy and automated image analysis following systematic shRNA-knockdown of our 27 candidate genes (Fig. 3b). Based on stringent criteria (see “Methods”), six genes significantly modified Parkin translocation (Fig. 3c and d; Additional file 2: Figure S2; Additional file 1: Table S5 and Table S6), including four genes (*GPATCH2L*, *PTCHD3*, *SVOPL*, and *ZNF543*) with consistent activities in both the mitochondrial morphology and Parkin translocation assays.

### Functional prioritization: α-synuclein-mediated toxicity

A wealth of evidence also supports a central role for α-synuclein-mediated toxicity in PD pathogenesis. α-synuclein aggregates, termed Lewy bodies, are the defining disease pathology and α-synuclein gene (*SNCA*) mutations, locus multiplication, and promoter polymorphisms are associated with PD susceptibility [5]. Further, expression of α-synuclein in numerous animal models including in the fruit fly [39–41], *Drosophila melanogaster*, recapitulates features of PD-related neurodegenerative pathology. Transgenic expression of α-synuclein in the fly retina leads to neurotoxic changes [39] and is amenable for detection of genetic modifiers [42, 43]. Genetic manipulation of established PD susceptibility genes, including *PARK2* [44, 45] and *VPS35* [46], modulate α-synuclein toxicity in transgenic flies, similar to findings in mammalian models [44, 47]. We therefore hypothesized that LoF in homologs of novel PD genes may similarly enhance α-synuclein-induced retinal degeneration.

**Fig. 4:**
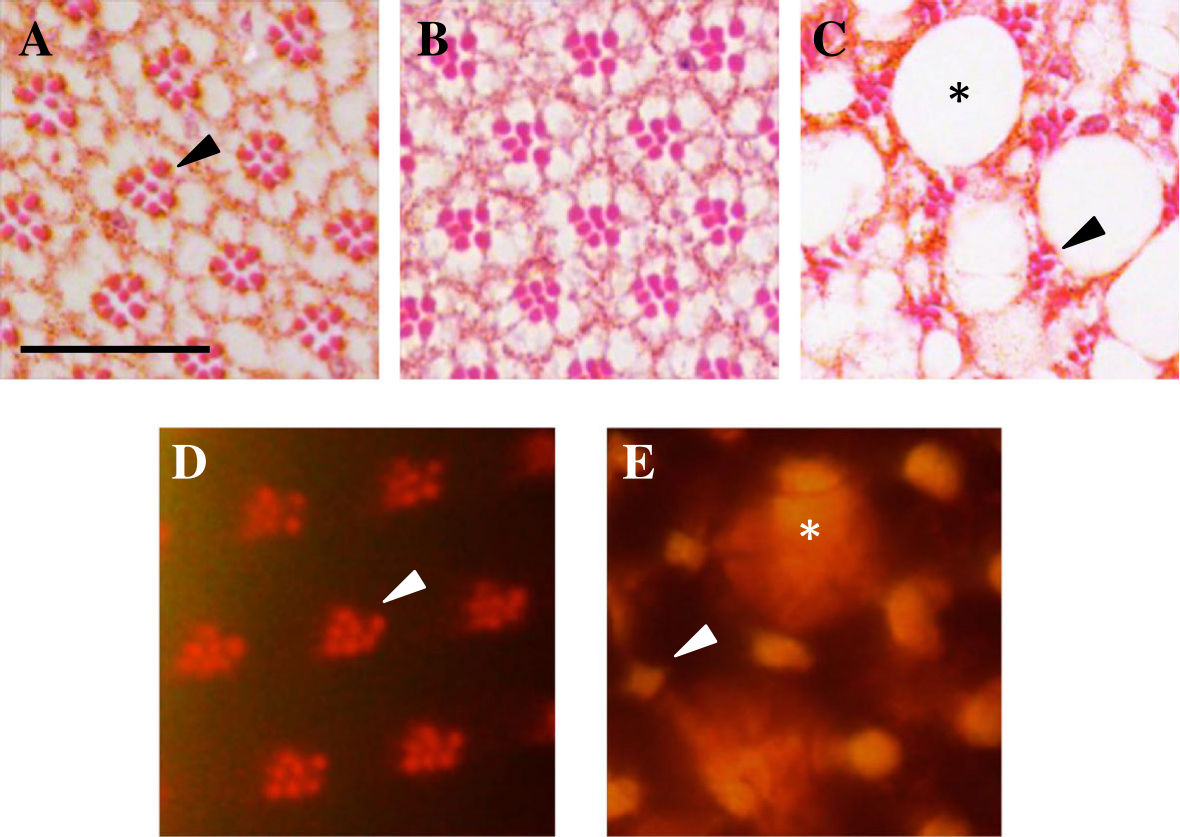
α-synuclein-induced retinal degeneration and screening assays in *Drosophila* transgenic animals*.* Tangential sections through the fly retina stained with hematoxylin and eosin reveal the ordered ommatidial array in control animals (**a** *Rh1-GAL4 / +*). Each ommatidia consists of a cluster of eight photoreceptive neurons (seven visible at the level examined). The photoreceptors each contain a single rhabdomere, the specialized organelle subserving phototransduction, giving the ommatidia cluster its characteristic appearance (arrowhead). Expression of *α-synuclein* in adult photoreceptors (**b**, **c** *Rh1-GAL4 / +*; *UAS-α-synuclein / +*) causes age-dependent, progressive retinal degeneration. Compared to one-day-old *Rh1 > α-synuclein* flies (**b**), histologic sections in 30-day-old animals (**c**) demonstrate rhabdomere/cell loss and substantial vacuolar changes (*asterisk*). The pseudopupil preparation allows visualization of rhabdomeres (*arrowhead*) in intact, unfixed intact fly heads, permitting medium-throughput screening for progression of *α-synuclein-*induced retinal pathology. Compared to controls (**d** *Rh1-GAL4 / +*), in 30-day-old *α-synuclein* transgenic animals (**e** *Rh1-GAL4 / +*; *UAS-α-synuclein / +*) rhabodomeres frequently appear indistinct (*arrowhead*) and vacuolar changes disrupt light refraction (*asterisk*). Representative control histology (**a**) and pseudopupil images (**d**) are shown for 15-day-old animals, the timepoint used for screening, in order to facilitate comparison with Fig. 5. Scale bar: 20 μm

Out of the 27 candidate genes implicated by our WES analyses, 13 were well-conserved in *Drosophila* (Additional file 1: Table S7). Available RNAi stocks targeting each of the 18 fly homologs (some genes had multiple conserved paralogs) were crossed to flies in which the human α-synuclein transgene was directed to adult photoreceptors using the *Rhodopsin1-GAL4 (Rh1)* driver (*Rh1 > α-synuclein*) [48]. For rapid screening, retinal neurodegeneration was monitored using the optical neutralization technique which allows assessment of retinal tissue integrity in intact, unfixed heads. In *Rh1 > α-synuclein* animals, the retina appears morphologically normal at 1 day (Fig. 4), but demonstrates age-dependent degeneration leading to progressive vacuolar changes, rhabdomere loss, and culminating with extensive tissue destruction by 30 days. At the 15-day time point selected for screening, only mild, if any, retinal pathology is detectable on most histologic sections, consistent with a weakly penetrant degenerative phenotype following optical neutralization (mean penetrance ~25%) (Fig. 4). However, co-expression of RNAi targeting fly homologs of four candidate genes (*ARSB*, *TMEM134*, *PTPRH*, and *VPS13C*) was observed to robustly enhance α-synuclein-mediated neurodegeneration in the retina (mean penetrance ~ 75%; Additional file 1: Table S8).

**Fig. 5:**
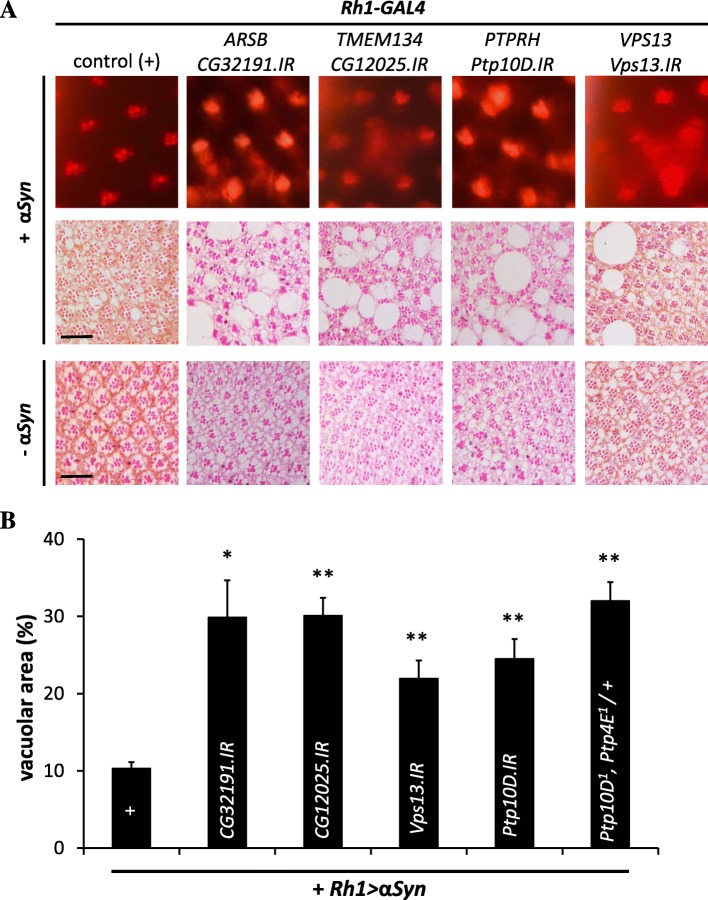
PD gene candidates harboring LoF variants enhance α-synuclein toxicity in *Drosophila*. Conserved fly orthologs of human genes discovered from WES analysis were targeted with RNAi (IR) and screened for enhancement of α-synuclein pathology using the pseudopupil assay (**a** *top row*). For each line evaluated, the severity of retinal degeneration was scored based on penetrance of the α-synuclein pseudopupil phenotype and enhancers required consistent results for at least two independent RNAi lines (see Additional file 1: Table S8). Representative results from the primary screen are shown for controls (*Rh1-GAL4 / +*; *UAS-α-synuclein / +*) and one IR line each for the implicated enhancers [Human Gene-Fly Ortholog (experimental genotype shown)]: *ARSB-CG32191* (*Rh1-GAL4 / +*; *UAS-α-synuclein / UAS-CG32191.IR.v14294*), *TMEM134-CG12025* (*Rh1-GAL4 / UAS-CG12025.IR.v104336*; *UAS-α-synuclein / +*), *PTPRH-Ptp10D* (*Rh1-GAL4 / UAS-Ptp10D.IR.v1102*; *UAS-α-synuclein / +*), and *VPS13-Vps13* (*Rh1-GAL4 / UAS-Vps13.IR.HMS02460*; *UAS-α-synuclein / +*). At the 15-day-old time point, *Rh1 > α-synuclein* causes a weakly-penetrant pseuodopupil phenotype and mild histopathologic changes which are amenable to modifier screening (compare with Fig. 4, panels c and e). Enhancers identified in the primary screen were confirmed based on retinal histology (**a** *middle row*) and demonstrated increased tissue destruction and disorganization. Activation of RNAi was not associated with any significant retinal degeneration in the absence of α-synuclein co-expression (**a** *bottom row*, *Rh1-GAL4 / IR transgene*). Scale bars: 20 μm. **b** Enhancement of α-synuclein-induced retinal degeneration was quantified based on the extent of vacuolar changes (area occupied by vacuoles / total retinal area). For quantification, three animals were examined per genotype. For *PTPRH*, additional confirmation was obtained by evaluating flies doubly heterozygous for strong alleles of the paralogs *Ptp10D* and *Ptp4e* (see also Additional file 2: Figure S5). Statistical comparisons were made using unpaired *t*-tests. *Error bars* are based on Standard Error of the Mean. *p < 0.05; **p < 0.01

All candidate enhancers of α-synuclein identified using the screening assay were further confirmed based on retinal histology, demonstrating accelerated pathologic changes with a significantly increased overall extent and severity of degeneration compared to *Rh1 > α-synuclein* controls without RNAi transgenes present (Fig. 5). Importantly, when each of these genes were targeted under similar experimental conditions (*Rh1 > RNAi*), but independent of α-synuclein expression, we did not observe any significant retinal pathology in 15-day-old animals (Fig. 5). Therefore, within the *Drosophila* α-synuclein transgenic model system, the implicated LoF enhancers appear consistent with synergistic (non-additive) effects on α-synuclein-mediated retinal degeneration. Since increased α-synuclein expression levels are one important mechanism of PD susceptibility [5], western blot analyses were performed to determine whether any of the identified genetic enhancers alter α-synuclein protein levels. However, following RNAi-mediated knockdown, none led to significant changes (Additional file 2: Figure S3). Thus, we hypothesize potential interactions with more downstream mechanisms of α-synuclein neurotoxicity. For 3 out of 4 candidate enhancers (*ARSB*, *VPS13C*, *PTPRH*), available siRNAs permitted additional testing of gene homologs as candidate modifiers in an established *C. elegans* model of α-synuclein toxicity [49]. However, no significant differences were detected in the α-synuclein-induced locomotor phenotype observed in one-week-old worms following knockdown of these genes (Additional file 2: Figure S4). We speculate that these contradictory results might stem from differences in assay sensitivity and/or tissue-specific toxic mechanisms as the fly and worm models are based on α-synuclein expression in the retina versus muscle, respectively.

Of the four genes discovered to interact with α-synuclein toxicity in *Drosophila*, we were able to obtain additional genetic reagents, including classical LoF alleles, for the two homologs of *PTPRH*: *Ptp10D* and *Ptp4E*. In our screen, two independent RNAi lines targeting *Ptp10D* robustly enhanced α-synuclein toxicity, but only one of the two available lines for *Ptp4E* met our threshold criteria (Additional file 1: Table S8). Interestingly, prior studies in *Drosophila* suggest that *Ptp10D* and *Ptp4E* are the result of a gene duplication event and these genes show evidence of partial functional redundancy, including for nervous system phenotypes [50]. Consistent with this, we found that transheterozygosity for strong (null) alleles of both genes enhanced α-synuclein-induced retinal degeneration (*Ptp4E* ^*1*^, *Ptp10D* ^*1*^ */ +*; *Rh1-Gal4 / +*; *UAS-α-synuclein* / +); whereas heterozygosity for either allele in isolation showed no significant enhancement (Fig. 5b and Additional file 2: Figure S5).

### Genetic replication of candidate PD genes from WES

We next evaluated our 27 gene candidates in additional available genetic datasets including: (1) an independent exome sequencing dataset from the Parkinson Progression Markers Initiative (PPMI) project [51]; (2) a whole-genome sequencing dataset including PD index cases of a Dutch genetic isolate belonging to the Genetic Research in Isolated Population (GRIP) program [52]; (3) an independent NeuroX exome array dataset [7, 53]; and (4) a large PD GWAS dataset [53]. Within the PPMI exome dataset, including 462 PD cases and 183 controls, evidence supporting replication was discovered for two genes, in which we identified the identical variants from the IPDGC discovery exome dataset (Additional file 1: Table S9). A PD case from PPMI carries the same homozygous stopgain variant (p.R362X) in *GPATCH2L* as observed for an IPDGC case. Although the age of onset differs 20 years between these two PD cases (47 and 68 years for the IPDGC and PPMI patients, respectively), they share similar asymmetric clinical symptoms at onset, which are characterized by resting tremor, bradykinesia, and rigidity. Furthermore, both PD cases have a father diagnosed with PD, implying the variant to be highly penetrant. We excluded the possibility that these two PD cases might be related by computing pairwise genetic relationships [54] from common SNPs (MAF ≥ 0.01). No evidence of relatedness was observed (A_jk_ = −0.0018). Based on ExAC, only one (0.003%) out of 32,647 European individuals has this same homozygous variant. The observation of two PD cases (0.12%) of our 1610 studied PD patients (1148 IPDGC WES plus 462 PPMI WES) with this *GPATCH2L* mutation is consistent with a 40-fold enrichment in our PD cohort. The second gene harboring an identical LoF variant is *FAM83A.* The p.G86X variant in *FAM83A*, detected within an IPDGC participant with sporadic PD diagnosed at the age of 28 years, was also observed in a single sporadic PD case from PPMI with an age of onset of 62 years. These *FAM83A* carriers presented with similar symptoms, including bradykinesa, rigidity, and resting tremor. In both datasets, the p.G86X allele is predicted to be in *trans* with another variant: p.R347X or p.V137G in PPMI and IPDGC, respectively.

The second genetic independent dataset that was investigated included a whole-genome sequencing study (39 PD index cases and 19 controls) of a genetic GRIP isolate from the Netherlands, focusing on variants within our candidate genes that were present in at least two PD index cases and absent in controls. We identified a heterozygous missense variant (NM_001127444:c.1176G > T:p.L392F) in *CD36* for three PD index cases. Although not consistent with a recessive inheritance model, this variant has not been observed in the 60,706 unrelated individuals of the ExAC database, suggesting potential enrichment in PD cases. These heterozygote variant carriers have a substantial higher age of onset (range, 61–79 years) in comparison to the PD patient (age of onset, 38 years) with the putative compound heterozygous variant within the discovery WES dataset. This observation supports an additive model of pathogenicity, implying more severe disease onset when two alleles are affected. Further, *CD36* (p.L392F) is predicted to represent the top 1% most harmful variants within the genome (CADD score = 23.3). In the IPDGC discovery dataset, the discovered compound heterozygous variants, p.Q74X and p.P412S (Table 1), are also predicted to be strongly deleterious (CADD scores of 26.5 and 25.9, respectively).

We next interrogated the independent IPDGC NeuroX dataset, including genotypes from 6801 individuals with PD and 5970 neurologically healthy controls. NeuroX is a genotyping array that includes pre-selected exonic variants and is therefore not suitable to search for the identical recessive LoF variants implicated by our WES analyses. Instead, we examined the burden of multiple variant classes within the 27 candidate genes, following the same variant categories as for the original IPDGC WES dataset (Additional file 1: Table S10). When only considering variants predicted to be deleterious (CADD > 20), an association is detected for *UHRF1BP1L* with PD risk (*p* = 0.005). This gene also shows an association with PD in the IPDGC WES dataset when performing a similar burden analysis considering missense variants (see above, *p* = 0.016). Using the NeuroX dataset, we additionally confirmed the enrichment of rare *PTPRH* variants in participants with PD (WES: *p* = 0.034, NeuroX: *p* = 0.045). Furthermore, *VPS13C* and *ARSB* show significant associations to PD when considering the joint effect of all variants, both common and rare (Additional file 1: Table S10).

Leveraging available IPDGC GWAS data (13,708 cases/95,282 controls), we next assessed for potential common variant association signals (*p* < 1 × 10^−4^) using a 1-Mb genomic window centered on each of the 27 candidate genes. Three loci (*VPS13C*, *PCDHA9*, and *TCHHL1*) showed evidence consistent with an association peak (Additional file 2: Figure S6). A genome-wide significant association at the *VPS13C* locus, was in fact recently reported [7]; the best SNP (rs2414739, *p* = 3.59 × 10^−12^) maps ~150 kb distal to *VPS13C*. Based on local patterns of linkage disequilibrium defined by Hapmap (Additional file 2: Figure S6), it is unlikely that rs2414739 is a proxy for p.E3147X or similar LoF variants in *VPS13C*; however, it might be possible that the SNP influences *VPS13C* expression by affecting the long non-coding RNA lnc-VPS13C-1 [55] in which the SNP is located. The other two candidate association peaks, adjacent to *PCDHA9* and *TCHHL1*, are considerably weaker signals (rs349129 = 1.40 × 10^−5^ and rs7529535 = 7.66 × 10^−5^, respectively) and given the distances (~500 kb) many other candidate genes are potentially implicated.

In sum, we identify additional genetic evidence consistent with replication for seven genes (*GPATCH2L*, *FAM83A*, *CD36*, *UHRF1BP1L*, *PTPRH*, *ARSB*, and *VPS13C*) that were implicated by our WES analysis, of which five (*GPATCH2L*, *UHRF1BP1L*, *PTPRH*, *ARSB*, and *VPS13C*) are further validated based on functional evidence from PD-relevant experimental models.

### Transcriptomics-based functional exploration

Lastly, we examined each candidate gene from our WES analysis for co-expression with established PD susceptibility gene in expression networks derived from human substantia nigra, leveraging available data from the United Kingdom Brain Expression Consortium (UKBEC) and the Genotype-Tissue Expression project [56]. Of the 27 candidate genes, seven were not sufficiently expressed in substantia nigra on the basis of UKBEC. Except for *DIS3*, these genes were also expressed poorly in publicly available data of the Genotype-Tissue Expression (GTEx) project [56]. Consequently, expression values for these genes were not used for construction of the UKBEC gene co-expression network (GCN). The remaining 20 genes were assessed for co-expression with known Mendelian PD genes (*ATP13A2*, *FBXO7*, *LRRK2*, *PARK2*, *PARK7*, *PINK1*, *RAB39B*, *SNCA*, and *VPS35*) using the UKBEC GCN (Additional file 1: Table S11 and Additional file 2: Figure S7). This approach highlighted three genes (*UHRF1BP1L*, *GPATCH2L*, and *PTPRH*) and the implicated networks were further interrogated based on gene set enrichment analysis using gene ontology (GO) terms to denote potential functions. *UHRF1BP1L* was co-expressed with *SNCA*, *PINK1*, *GBA*, and *ATP13A2* in a network significantly enriched for genes with roles in synaptic transmission (*p* = 2.27 × 10^−11^) as well as astrocytic (*p* = 8.18 × 10^−8^) and dopaminergic neuronal markers (*p* = 3.98 × 10^−46^). *GPATCH2L* was co-expressed with *PARK7* in a network enriched for other neuronal genes (*p* = 3.41 × 10^−12^) with cellular roles in metabolism of macromolecules (*p* = 3.82 × 10^−15^). Lastly, *PTPRH* was assigned to a co-expression module including *FBX07* and enriched for oligodendrocyte markers (*p* = 8.69 × 10^−22^). Importantly, the implicated modules were preserved (Z.summary > =10) in the independent GTEx dataset.

## Discussion

We report the results from WES analysis in the largest PD cohort studied to date. Assuming a recessive inheritance model, we identified 27 candidate genes harboring rare homozygous or compound heterozygous LoF variants. With the exception of *ARSB*, we did not identify recurrent recessive alleles in more than a single PD case. This result—potentially consistent with a highly heterogeneous genetic etiology for PD—creates significant barriers for statistical confirmation and genetic replication of novel PD susceptibility loci. Additional genetic samples were not available for segregation analysis and given the rarity and heterogeneity of the implicated alleles, definitive human genetic replication would likely require very large sample sizes, including many thousands of PD cases with either WES or gene resequencing. We therefore coupled our WES analyses with functional studies in both mammalian cells and experimental animal models, including *Drosophila* and *C. elegans*, in order to prioritize genes for future study. Our results highlight 15 out of the 27 gene candidates that interact with mitochondrial dynamics and five loci that enhance α-synuclein-mediated neurodegeneration. As discussed below, while these results highlight a promising subset of genes with potential links to PD-relevant mechanisms, we cannot exclude contributions from other implicated genes/variants. All of these data, including promising variants from the human genetic analyses and results of functional studies, will be a valuable resource for future investigations of PD genomics. Analyses of several other WES and complementary large-scale, genetic datasets provide additional evidence supporting replication for 7 out of 27 genes. Evidence from human genetics and functional studies converge to most strongly implicate five gene candidates discussed below; however, further investigation will be required to definitively link each of these loci to PD susceptibility and elucidate the relevant mechanisms. Nearly all of these genes are robustly expressed in brain [56], including the substantia nigra, thereby consistent with their implication in PD. A subset (*GPATCH2L*, *UHRF1BP1L*, and *PTPRH*) are co-expressed with established Mendelian PD genes in the substantia nigra based on analyses of UKBEC and GTEx expression data. In sum, our results define several promising new susceptibility loci candidates for further investigation and illustrate a powerful, integrative discovery strategy for future, large-scale PD genomic studies.

Mitochondrial mechanisms have been strongly implicated in PD risk and pathogenesis [28, 30]. Following shRNA-mediated knockdown, 15 candidate recessive loci identified in our WES dataset showed effects on mitochondrial morphology and Parkin translocation to mitochondria in cell culture. We focus our initial discussion on three genes, *GPATCH2L*, *UHRF1BP1L*, and *VPS13C*, for which we discovered additional genetic evidence consistent with replication in independent cohorts. In the IPDGC cohort, a single PD case was identified with a homozygous stopgain variant (p.R362X) in *GPATCH2L* and a second individual with the identical, rare genotype was discovered in PPMI. This variant is reported with a low frequency of 0.003% in ExAC. Although minimal clinical or demographic information is available within ExAC, this finding is compatible with population prevalence estimates for PD [20]. Nevertheless, genotyping of p.R362X in additional large PD case and control cohorts will be required to definitively establish an association with PD susceptibility. *GPATCH2L* knockdown both increased mitochondrial roundness and impaired Parkin translocation. The encoded protein, GPATCH2L, which has not previously been studied, contains a glycine-rich RNA-binding motif, the “G-patch” domain [57]. *GPATCH2*, a paralog of *GPATCH2L*, is upregulated in cancer cells, localizes to the nucleus where it interacts with RNA-processing machinery, and manipulation in culture alters cell proliferation [58, 59]. Notably, *GPATCH2L* is non-conserved in either the *C. elegans* or *Drosophila* genomes, precluding study of this candidate in these models. While our results using cellular assays implicate *GPATCH2L* in mitochondrial quality control mechanisms, further follow-up studies in mammalian model systems will be needed to confirm a role in PD pathogenesis.

Another promising gene, *UHRF1BP1L*, harbored a homozygous stopgain variant (p.K1376X) in a single IPDGC case. This is a novel variant, based on its absence from the ExAC cohort. Additional support for *UHRF1BP1L* as a *bona fide* PD locus comes from complementary analyses in both the IPDGC WES and NeuroX datasets, documenting a burden of rare missense and LoF variants in association with disease risk. In the UKBEC, *UHRF1BP1L* was associated with a substantia nigra co-expression module including both *SNCA* and *PINK1*, reinforcing potential links with established PD genetic mechanisms. Indeed, *UHRF1BP1L* knockdown cause sharply reduced mitochondrial numbers and altered morphology. Interestingly, *UHRF1BP1L* encodes a protein bearing an amino terminal homologous to yeast VPS13 and studies in cell culture provide support for a role in retrograde transport from the endosome to the trans-Golgi network [60].

Notably, LoF in human *VPS13C* was also implicated by our analyses of IPDGC WES data and knockdown disrupted mitochondrial morphology. Besides the single IPDGC case, several families with autosomal recessive early onset Parkinsonism and dementia due to *VPS13C* were recently reported [21] and this locus also harbors common PD susceptibility variants based on GWAS [7]. Our findings of a potential mitochondrial role for *VPS13C* agree with those of Lesage et al. who additionally reported that VPS13C localizes to the outer membrane of mitochondria and LoF was associated with reduced mitochondrial membrane potential, fragmentation, and increased Parkin-dependent mitophagy. Importantly, *VPS35*, which causes autosomal dominant, late-onset PD, is similarly involved in endosomal trafficking [61] and has also recently been implicated in mitochondrial dynamics [62], including interactions with Parkin [63]. Like *UHRF1BP1L*, *VPS13C* and *GPATCH2L* are expressed in the brain, including within the substantia nigra; however, additional work will be needed to define their functions, including potential interactions with other established disease genes (e.g. *VPS35*, *parkin*) and requirements for mitochondrial maintenance.

Based on functional screening in *Drosophila*, four candidate genes from our WES analyses were implicated as LoF enhancers of α-synuclein neurotoxicity, which also has a central role in PD pathogenesis. We discuss the three genes (*VPS13C*, *PTPRH*, and *ARSB*) where additional human genetic evidence supports replication. Interestingly, besides its requirement for mitochondrial maintenance, RNAi-mediated knockdown of *Drosophila Vps13* enhanced α-synuclein toxicity. In the single reported *VPS13C* PD case with a completed autopsy, neuropathological findings included abundant α-synuclein aggregates in both the brainstem and cortex [21]. Thus, *VPS13C* and associated endosomal sorting pathways (including *VPS35*) may represent a point of convergence for mitochondrial and α-synuclein-mediated PD mechanisms. Consistent with this, evidence for the impact of α-synuclein toxicity on mitochondria has recently emerged [28], including from studies in mammals [64].

In the IPDGC WES cohort, a single PD case was discovered with compound heterozygous LoF variants in *PTPRH* (p.Q887X and p.E200X). Both variants were also observed at low frequencies in the ExAC database (0.039% and 0.003%, respectively); however, they each met our pre-specified threshold of < 1% based on the population prevalence of PD. Encoding a receptor protein tyrosine phosphatase, *PTPRH* (also called *SAP-1*) was first discovered for its potential association with gastrointestinal cancers [65, 66] and remains poorly studied in the nervous system context. In studies of both vertebrates and invertebrates, receptor protein tyrosine phosphatases have been strongly implicated as key neural cell adhesion receptors, with roles in neurodevelopment and synaptic function, and other members of this family have been implicated in numerous neuropsychiatric disorders [67]. In *Drosophila*, RNAi-mediated knockdown of the conserved *PTPRH* ortholog, *Ptp10D*, enhanced α-synuclein-triggered retinal degeneration, but was not associated with substantial neurotoxicity independent of α-synuclein expression. *Ptp10D* mutant flies are also viable and fertile but demonstrate long-term memory deficits in behavioral assays [68]. More recent studies further implicated Ptp10D in neural-glial interactions during development of the central nervous system [69], potentially consistent with our findings that human *PTPRH* participates in a substantia nigra gene co-expression network strongly enriched for oligodendrocyte markers. Besides our discovery of homozygous LoF in *PTPRH*, further analyses of the IPDGC WES dataset, and the substantially larger, independent NeuroX cohort, implicate a burden of rare variants at this locus in association with PD susceptibility.

α-synuclein-induced neurodegeneration was also enhanced by knockdown of *CG32191*, a *Drosophila* homolog of *ARSB*. RNAi transgenic lines targeting three other conserved fly *ARSB* homologs showed consistent interactions with α-synuclein (Additional file 1: Table S7 and Table S8). In the IPDGC cohort, we discovered four PD cases homozygous for a variant predicted to disrupt splicing of exons 1 and 2 in *ARSB*. Although the identified variant has not previously been documented in ExAC, we identified a single IPDGC control homozygote. Additional evidence supporting association of the *ARSB* gene with PD susceptibility comes from burden analysis in the independent NeuroX cohort. The surprisingly common *ARSB* splicing variant (rs138279020, MAF = 0.065 in IPDGC) is a single nucleotide insertion allele within a poly-A repeat, which we speculate might lead to inefficient capture in prior WES and possibly explain the absence of this variant from ExAC and the 1000 Genomes project reference. All four PD cases in our data with the homozygous *ARSB* splicing variant were confirmed by Sanger sequencing. Intriguingly, mutations in *ARSB*, encoding the lysosomal enzyme Arylsulfatase B, are associated with the recessive lysosome disorder, Mucopolysaccharidosis type VI (MPS VI, also called Maroteaux-Lamy syndrome), in which the glycosaminoglycan, dermatan sulfate, accumulates causing skeletal dysplasia and other heterogeneous manifestations [70]. Substrate accumulation and associated cellular stress has been reported to induce markers of impaired autophagy and mitochondrial dysfunction in *ARSB* deficient fibroblasts from MPSVI patients, as in other lysosomal disorders [71, 72]. Importantly, Maroteaux-Lamy can be characterized by minimal or even absent clinical signs, leading to incidental discovery or diagnosis in adulthood, and such mild phenotypes have been suggested to accompany partial LoF with preserved low-level ARSB enzymatic activity [70, 73, 74]. Similar genotype–phenotype relationships have been documented for other lysosomal-storage disorders, including Gaucher’s disease, which has established links with PD risk [75, 76]. While a full accounting is outside the scope of this study, at least one of the three IPDGC cases for which records were available revealed clinical features potentially overlapping with MPS VI.

The strengths of our study include the largest PD WES discovery dataset assembled to date, complementary analyses in independent available cohorts to establish replication, and integration of promising human genetic findings with multiple functional assays relevant to PD mechanisms. Nevertheless, we also make note of several inherent limitations. In order to prioritize candidate genes for initial investigation, assumptions were made concerning the specific inheritance model (recessive) and stringent criteria were employed for variant filtering. In the future, it will be important to also consider the possibility of dominantly acting alleles; however, this substantially increases the number of variants to consider and also potentially complicates functional studies (i.e. compared with LoF screening using RNAi). Our study design excluded consideration of many non-synonymous variants that could potentially cause loss (or gain) of gene function, along with certain non-truncating, frameshifting alleles (see “Methods”). Even with fairly stringent criteria for variant filtering and the assumption of recessive inheritance, we found evidence for substantial etiologic heterogeneity. Improved confidence for the discovery of PD causal variants will likely come from PD WES cohorts with significantly enhanced sample sizes, as well as increased numbers of adult controls, including those with careful neurological assessments to exclude mild PD symptoms. Indeed, most of the variants implicated by the IPDGC WES cohort were represented at low frequencies within the largest available public database, ExAC [77, 78]; however, we have no information about potential PD manifestations in such individuals or even participant age.

Since no single cellular or animal experimental model is expected to universally recapitulate all potential facets of disease biology, we note that the employed functional screening assays are potentially liable to false-negative or false-positive findings. Importantly, experimental evidence of a genetic interaction with either mitochondrial dynamics or α-synuclein-mediated neuronal injury in our screening assays cannot in isolation confirm a role in disease causation, but rather serves to prioritize genes for future investigation. Out of the 27 candidate genes implicated in the IPDGC WES discovery analysis, 14 were insufficiently conserved for follow-up in α-synuclein transgenic flies. While simple animal models, including *Drosophila* or *C. elegans*, have made important contributions to our understanding of PD pathogenesis, selected mechanisms, such as the potential role of adaptive immunity or basal ganglia circuit dysfunction, cannot be addressed in invertebrates [79, 80]. We were unable to confirm our findings from *Drosophila* in a published *C. elegans* model of α-synuclein toxicity. In the future, it will also be important to examine potential genetic interactions in other PD models, including *LRRK2* transgenic flies or those containing mutations in other PD loci, such as *VPS35* or *parkin*. While neuroblastoma cells offer the convenience of robust mitochondrial readouts, they are limited by their undifferentiated, transformed state distinct from that of postmitotic neurons. In the future, human-induced pluripotent stem cells, including those derived from individuals with PD, can be differentiated into dopaminergic or other neuronal types and potentially deployed for functional screening strategies. Additionally, genome-editing technologies may facilitate systematic functional evaluation of candidate disease-associated variants of unknown significance.

## Conclusions

We have identified five excellent PD gene candidates (*GPATCH2L*, *UHRF1BP1L*, *PTPRH*, *ARSB*, and *VPS13C*), harboring homozygous or compound heterozygous LoF variants in PD exomes, demonstrating functional interactions with mitochondrial and/or α-synuclein-mediated mechanisms, and supported by evidence of replication in independent human datasets. The recent report [21] of additional PD families segregating LoF mutations in *VPS13C* along with other experiments supporting a role in mitochondrial mechanisms significantly strengthens the evidence in support of this gene in PD and validates our overall approach. These loci are well-suited for future efforts directed at human genetic replication and in-depth functional dissection. We also make available results, including findings from human genetic analyses and functional studies in most cases, on 22 other promising loci. These data will serve as a valuable reference for ongoing and future PD genetic studies. More broadly, our approach of integrating high-throughput sequencing in PD case/control cohorts with parallel systematic screening in cells and model organisms for functional prioritization exemplifies a powerful experimental strategy with great promise for future genomic studies of PD and other human disorders.

## Methods

### Genetic analyses

#### Whole-exome sequencing

WES was performed on 1148 PD cases and 503 neurologically healthy controls of European descent. All participants provided written informed consent. Relevant local ethical committees for medical research approved participation in genetic studies. If PD patients were prescreened for known pathogenic mutations, they were excluded for exome sequencing when having such a variant. The cases were diagnosed with PD at a relatively young average age of 40.6 years (range, 6–56 years), of which approximately 37% reported a positive family history. The neurologically healthy controls are on average 48.2 years of age (range, 10–97 years). A more extensive overview of demographic information is reported in Additional file 2: Figure S8.

Due to improvements of the exome sequencing protocol over time, the exome sample libraries were prepared with different capture kits. For this study, three different capture kits were used: Illumina TruSeq (San Diego, CA, USA) (62 Mb target); Roche (Basel, Switzerland) Nimblegen SeqCap (44.1 Mb target); and Agilent (Santa Clara, CA, USA) SureSelect (37.6 Mb target), which captured 96%, 81%, and 71% of the targeted exome at least ten times, respectively (Additional file 1: Table S12). Exome libraries were sequenced on a HiSeq 2000 (Illumina, San Diego, CA, USA). The Burrows Wheeler Aligner MEM v0.7.9.a [81] was used to align the 100-bp paired-end reads to the human reference genome build hg19. We called the single nucleotide variants (SNVs) and insertions/deletions (indels) for all samples simultaneously using Genome Analysis Toolkit (GATK) 3.x [82], followed by the exclusion of low-quality variant calls not passing the default GATK filters. Individual genotypes were removed with genotype quality Phred-scores below 40. ANNOVAR [83] was applied to annotate the variants with information concerning variant type (valid annotations when Refseq in concordance with UCSC), MAF in the general population, and predictions of the variant’s effect on gene function, implementing CADD [84].

#### Variant identification in IPDGC WES dataset

Considering the worldwide prevalence of 0.041% for PD in the age range of 40–49 years [20], we selected rare variants with a MAF < 1% (corresponding to a homozygous frequency of 0.01%) in the European population. Because the specified 0.041% of the population with young-onset Parkinson’s disease (YOPD) is not caused by one shared genetic factor, we expect a homozygous frequency of 0.01% to be an adequate cutoff, which would be able to determine variants present in approximately 25% of the YOPD population. As a comparison to the most common genetic cause of YOPD, *parkin* [85], the most frequent mutation is an exon 3 deletion, which has been identified in 16.4% of YOPD patients [86]. Using ANNOVAR [83], all variants were annotated with MAF information of ESP6500si (European American population) [87], 1000 Genomes Project (European population of April 2012 version) [88], and the ExAC browser (non-Finish European population) [77, 78]. When no public allele frequency was available for homozygous variants, the in-house control dataset of 503 individuals was used as a reference for the general population. Homozygous variants were excluded when being common (>1%) in controls or having a relative higher frequency in controls than in cases. KGGseq [89] was used to count the number of homozygous variants for the cases versus controls.

In addition to the population allele frequency filters, we only selected SNVs and indels affecting the position of the stop codon or located at a splice site (within 2 bp of splicing junction), which are variants expected to result in a loss of gene function. As the aim of this study was to validate our approach to identify high promising PD candidate genes, rather than discovering all putative PD genes present within our WES dataset, we set a conservative selection criteria by only including frameshifts that caused an immediate stopcodon at the position of the indel. Splice-site variants were only considered when being adjacently located to an exon that is coding for amino acids. As a final filter for the homozygous variants, we manually excluded variants that failed GATKVQSR and hard filtering. Quality predictions based on the ExAC database are more adequate, as it includes ~37× more samples than our dataset.

For the putative compound heterozygous mutations, both variants should be located within the same transcript and at least one allele should contain a LoF variant. The second variant could be: (1) a LoF variant; or (2) a missense variant that is absent in dbSNP137 [90] database and with a CADD score > 20 (predicted to belong to the 1% most deleterious variants of the total genome), indicating a pathogenic effect. The latter two filter criteria should decrease the chance of including benign missense variants. The putative compound heterozygous variants were identified by scoring the number of variants per sample per gene with PSEQ (https://atgu.mgh.harvard.edu/plinkseq/pseq.shtml). The reads of variants located within approximately 200 base pairs were visualized in IGV [91] to judge the authenticity of the compound heterozygous variant. When the different variants are located on distinct alleles, the combination of variants was considered a true compound heterozygous mutation.

All recessive variants that remained after the filtering procedures were Sanger sequenced to confirm the variant calls generated by the exome pipeline.

#### Variant aggregation analyses in the IPDGC WES dataset

SKAT-c [92] was used to analyze the burden of coding variants for each identified gene. Both rare variants only and the joint effect of common and rare variants were tested. Because variant aggregation tests are prone to coverage differences, capture usage and population stratification, we performed a more stringent individual and variant QC, resulting in a reduced dataset of 1540 samples (1062 cases and 478 controls) covering 268,038 variants. Individuals were excluded when failing gender test, showing evidence of relatedness, having dubious heterozygosity/genotype calls, or being a population outlier. Variants were removed when having a genotype missingness > 5%, a Hardy–Weinberg equilibrium *p* value < 1e^−6^ or a *p* value for non-random missingness by phenotype < 1e^−5^. Variants were only considered for association analyses if located in a region targeted by all different capture kits.

Benign variants have the potential to dilute a true association signal of the combined effect of functional variants in a gene. We therefore annotated variants with ANNOVAR [83] to group variants according to their type or predicted pathogenicity. Two subsets of variants were examined: (1) predicted pathogenic variants, including LoF variants and missense mutations that are predicted to be pathogenic by the CADD framework; and (2) missense variants, including amino-acid changing and LoF variants.

As suggested by SKAT, we selected a MAF cutoff of 0.018, which is based on the total sample size and separates rare and common variants. Common variants (MAF > 0.018) were pruned using PLINK [93] (indep settings 50 5 1.5). Due to confounding factors (usage different capture kits and multiple CEU populations), 20 principle components, 10× coverage, and gender were taken into account as covariates. Both a traditional one-sided burden (assuming all variants to have a harmful effect) and a two-sided SKAT test (allowing variants to be either damaging or protective) were performed. Empirical *p* values were calculated by comparison of the nominal *p* value to 10,000 permutations of affection status. Genes with an empirical *p* value < 0.05 were considered to be significantly associated to PD.

#### Genetic replication 1: variant identification in PPMI WES dataset

We obtained permission to access WES data generated by the PPMI [51]. After standard variant and individual QC, the dataset includes 477,512 variants for 462 PD cases and 183 neurologically healthy controls. A similar search for homozygous and putative compound heterozygous LoF variants, as described for the original IPDGC WES dataset, was applied for this second independent PPMI WES dataset by using ANNOVAR [83] and KGGSeq [89].

#### Genetic replication 2: GRIP genetic isolate

The southwest of the Netherlands contains a recently isolated population which is part of the GRIP program [52]. A total of 39 PD index cases and 19 controls of this isolate were subjected to whole-genome sequencing to explore the genetic factors underlying PD within this geographic region. Missense and LoF variants which were present in at least two index cases and a MAF < 0.1% in public databases (ExAC, 1000G dbSNP138, and ESP6500) were considered as potential PD variants. Genes harboring such variants were surveyed for overlap with our list of candidate genes.

#### Genetic replication 3: variant aggregation analyses in NeuroX

We investigated the genetic burden of common and rare variants in these genes by using the independent NeuroX dataset, which is generated by a custom-made genotype array [53] using a backbone of ~240,000 standard Illumina Exome content as a basis with an additional ~24,000 variants that are suggested to be involved neurological diseases. The same procedures as described for the burden test in the IPDGC WES dataset were applied. After QC, a total of 6801 PD cases and 5970 neurologically healthy controls remained with high-quality genotype data for 178,779 variants. Based on the sample size, the MAF cutoff was 0.0063.

#### Genetic replication 4: overlap PD risk loci

Approximately 70% of the participants included in this study have also been included in previous published GWAS [7, 94, 95]. To explore the possibility that our candidate genes might also contain common risk variants increasing the risk to develop PD, next to the identified LoF variants with assumed high penetrance, we searched for GWAS loci within 1 Mb upstream and downstream of the gene of interest using the recent PD meta-analysis through pdgene.org [7]. Significant associations and suggestive *p* values < 1e-4 were considered. To understand the underlying linkage disequilibrium structure, LocusZoom [96] was applied to visualize the European 1000G recombination events for the candidate genes that were closely located to a GWAS locus.

#### Gene co-expression analyses

We constructed gene co-expression networks (GCN) from two different substantia nigra datasets using the R software package, WGCNA (weighted gene co-expression network analysis) [97]. This was followed by the same post-processing of WGCNA gene modules based on k-means: a heuristic to rearrange misplaced genes between modules using the number of modules detected by the standard WGCNA as k and the eigengenes as centroids. The first GCN is based on 19,152 genes from 65 substantia nigra control brains from the UKBEC consortium. The gene expression profiles are based on Affymetrix Exon 1.0 ST Arrays [98]. The second GCN is based on 63 samples from the same tissue, GTEx [56] V6 gene RPKM values. Genes were filtered with a RPKM based cutoff of 0.2 and missingness < 30% resulting in the analysis of 18,363 Ensembl genes. We corrected this gene expression dataset for the principal components significantly correlated with GTEx samples covariates using the Swamp R package. WGCNA gene modules were functionally annotated with gProfileR [99] R software package using GO database, accounting for multiple testing with gSCS’s gProfiler test. Background genes used were all genes in the substantia nigra GCN. Cell type enrichment analysis was performed with the userListEnrichment function with brain specific enrichment, implemented in the WGCNA R package. Preservation analysis of UKBEC GCN in GTEx’s substantia nigra profiles was performed with WGCNA’s preservation analysis. Results are reported with the Z.summary statistic [100]. Graphical representation of the GCN subnetworks were constructed by using the 27 candidate genes and known PD genes (ATP13A2, FBXO7, LRRK2, PARK2, PARK7, PINK1, RAB39B, SNCA, and VPS35) as seed genes. For each of these genes sequentially, in a round robin fashion, we added the gene with highest adjacency, based on TOM values, and the links this gene has with all the seed genes. We used Cytoscape 3.3 for display with a Kamada-kawai layout algorithm [101].

### Human cellular screen

#### shRNA virus production

Bacterial glycerol stocks containing the shRNA vectors (Sigma, St. Louis, MO, USA; TRC1 and 1.5) were grown overnight in Luria-Bertani media containing 100 μg/mL of ampicillin (Sigma-Aldrich, St. Louis, MO, USA). We selected at least five shRNA clones per gene. Endotoxin-free shRNA plasmids were extracted according to the manufacturer’s protocol (Zymo, Irvine, CA, USA; ZR Plasmid Miniprep Classic kit). Lentivirus was produced as follows: HEK293T packaging cells were seeded at a density of 4 × 10^Δ5^/mL (100 μL per well) in cell culture media, Optimem (Invitrogen, Carlsbad, CA, USA) containing 10% fetal bovine serum (FBS) in 96-well tissue culture plates. Cells were incubated for 24 h (37 °C, 5% CO_2_). Each well was subsequently transfected with 100 ng of shRNA plasmid, 90 ng of packaging plasmid (pCMV-dr8.74psPAX2), and 10 ng of envelope plasmid (VSV-G/pMD2.G) combined with 0.6 μL of FugeneHD (Promega, Madison, WI, USA) in a total volume of 10 μL. Transfection efficiency was monitored using the pKLO.1 GFP plasmid (Sigma, St. Louis, MO, USA) and had to be greater than 90%. Sixteen hours after transfection, media was refreshed and supernatant harvested after a further 24 h. Virus was stored at −80 °C.

To ensure successful lentivirus production, HEK293T cells were plated out at a density of 2 × 10^Δ5^/mL (100 μL per well) in Optimem containing 10% FBS and 15 μg/mL of protamine sulfate (Sigma, St. Louis, MO, USA). Cells were infected with 10 μL, 25 μL, and 50 μL of lentivirus. The following day, media was refreshed with media containing 2.5 μg/mL of puromycin. After a further three days, plates were manually inspected to determine cell viability of each well. If more than 10% of the wells contained dead cells, lentiviral production for that plate was repeated.

#### Neuroblastoma cell culture

BE(2)-M17 (ATCC® CRL-2267™) and HEK 293 T (ATCC® CRL-3216™) cell lines were obtained from the American Type Culture Collection (Manassas, VA, USA). BE(2)-M17 cell lines were cultured in Dulbecco’s Modified Eagle/Nutrient Mixture F-12 Medium (DMEM/F-12) with GlutaMAX (Invitrogen, Carlsbad, CA, USA) supplemented with 10% FBS, 1× non-essential amino acids (NEAA), and 1% Penicillin/Streptomycin. HEK 293 T cells were cultured in Opti-MEM (Invitrogen, Carlsbad, CA, USA) containing 10% FBS and 1× NEAA. All cell lines were routinely tested for mycoplasma contamination. For lentivirus infection, 25 μL of the lentivirus was added to each well of a 96-well plates and protamine sulfate was added at a final concentration of 1 μg/mL in each well of the 96-well plate. Specific wells on each lentiviral plate contained GFP expressing virus to ensure efficient transduction.

#### Cell-based screening assays

Four phenotypes were studied in two different assays:

Mitochondrial morphology [33] was examined in a single assay with BE(2)-M17 cells, which were expanded and plated at a density of 5 × 10^Δ4^/mL (100 μL per well) in 96-well black CellCarrier plates (PerkinElmer, Waltham, MA, USA) pre-pipetted with 25 μL of the lentivirus. On day 2, media was refreshed with DMEM/F12 (with 10% FBS) supplemented with 2 μg/mL puromycin. On day 4, the cells were incubated with 100 nM MitoTracker Red CMXros, 100 nM MitoTracker DeepRed (Molecular Probes), and 1 μg/mL Hoechst for 20 min at room temperature. Media was refreshed and the cells were incubated for a further 2 h before fixation with 4% paraformaldehyde (pH 7.3).We examined three parameters commonly used for quantification of mitochondrial morphology: mitochondrial number, axial length ratio, and roundness.

For the Parkin translocation assay BE(2)-M17 cells were also utilized. The PLVX inducible vector (Clontech, Mountain View, CA, USA) overexpressing C-terminally tagged Parkin-GFP was used to make polyclonal stable BE(2)-M17 cells. Stable cell lines were cultured in DMEM/F12 supplemented with 10% FBS, 1% NEAA, 1% P/S, 250 ng/mL Puromycin, 200 μg/mL G418, and 1 μg/mL of doxycycline. BE(2)-M17 cells were expanded and plated at a density of 7.5 × 10^4/mL (100 μL per well) in 96-well black CellCarrier plates (PerkinElmer, Waltham, MA, USA) pre-pipetted with 25 μL of the lentivirus. The following day, media was exchanged with media without doxycycline to induce the expression of Parkin-GFP. On day 5, the cells were incubated with 100 nM MitoTracker DeepRed (Molecular Probes, Eugene, OR, USA) and 1 μg/mL Hoechst. After 20 min, media was refreshed with media containing 15 μM Carbonyl cyanide m-chlorophenyl hydrazone (CCCP). Cells were incubated for 2 h before fixation in 4% paraformaldehyde (pH 7.3).

#### Image acquisition and analysis

Image acquisition was carried out using the automated confocal imaging system, Cell Voyager CV7000 (Yokogawa, Tokyo, Japan). The mitochondrial morphology assay involved a total of 60 fields per well using a 60× water immersion objective lens for improved resolution. Nuclei were imaged utilizing the 405 nm laser, Mitotracker CMXros utilizing the 561 nm laser, and mitotracker DeepRed utilizing the 640nM laser. For the translocation assay, a total of 60 fields per well were taken using a 20× objective lens. Nuclei were imaged utilizing the 405 nm laser, Parkin-GFP utilizing the 488 nm laser, and mitotracker DeepRed utilizing the 640 nm laser.

Images were stored and analyzed by the Columbus Image Data storage (PerkinElmer, Waltham, MA, USA). Image quality control: only well-segmented interphase cells were included. Mitotic, apoptotic badly segmented, and out-of-focus cells were excluded. Cells touching the border of the image were removed to avoid analysis of artificially cropped cells. All wells where the perturbation strongly decreased cell number were disregarded. Morphological characteristics and signal intensities were quantified and results exported to R package CellHTS2. To quantify mitochondrial morphology, the median mitochondrial number per object, roundness, axial length ratio, and intensity of mitorackerCMXros (mitochondrial potential) were calculated.

To differentiate between CCCP-treated Parkin stable cell lines and untreated cells, the number of spots formed on mitochondria was calculated. Cells containing more than two spots were considered positive for Parkin translocation. The ratio of cells positive for translocation versus the number of cells negative for translocation was calculated per well to give a cell number independent measure of Parkin translocation. CCCP-treated cells transduced with a scrambled shRNA and CCCP-treated cells transduced with shRNA targeting *PINK1* were included on each plate. An average Z’ of 0.61 was calculated for the entire screen, with a minimum Spearman’s Rank correlation between replicates of 0.8.

Data from high content imaging assays were analyzed using the BioConductor CellHTS2 package for the R software environment (R version 2.11.1, BioConductor version 2.6). Data were normalized to negative controls on a per-plate basis to minimize plate-to-plate variation. For the Parkin-translocation screen, negative controls were considered as wells which had been transduced with lentivirus encoding a scrambled sequence and had been treated with CCCP. For the remaining screens, negative controls were considered as wells that had been transduced with lentivirus encoding a scrambled sequence.

#### Statistical analysis

For each of the shRNA screens, each assay plate was completed with six replicates to enable the detection of subtle effects and minimize false negatives. For each shRNA, Mann–Whitney U tests with false discovery rate (FDR) correction were performed and the robust strictly standardized median difference (SSMD*) was calculated [102]. Effects were considered significant when the SSMD* normalized effect of shRNA treatment was greater than or less than 4 or −4 and at least two independent clones per gene showed a significant effect. Seed sequences were manually inspected to ensure no common sequence.

For each assay, a positive control plate containing known modifiers of the phenotype in question was run in parallel to ensure the assay worked optimally. The robust Z-factor was calculated as previously described [103], using the normalized values for the controls from all plates. For the mitochondrial assay, known regulators of mitochondrial fission or fusion were included. For the Parkin translocation assay, TOMM7 and PINK1 were used as positive controls.

#### shRNA knockdown validation

Cell culture and shRNA mediated knockdown were performed as described above. Cells were harvested for RNA isolation using the SV 96 Total RNA Isolation System (Promega, Madison, WI, USA) according to the manufacturer’s protocol. Total RNA primed with oligo dT (Qiagen, Hilden, Germany) was used for cDNA synthesis with Superscript III RT (Life Technologies, Carlsbad, CA, USA) according to the manufacturer’s specifications. Quantitative polymerase chain reaction (PCR) was carried out in triplicates on a ViiA7 real-time PCR system using SYBR Green PCR master mix (Life Technologies, Carlsbad, CA, USA) and 0.04 μM specific primer pairs for all targets. For multiple exons, gene primers were designed to span exon-exon junctions or to be separated by one intron on the corresponding genomic DNA. Normalized relative quantities were calculated with HMBS as housekeeping gene by using the qbasePLUS software (Biogazelle, Gent, Belgium) and knockdown efficiencies per clone were calculated using scrambled control wells (*n* = 3) as a reference.

### Animal models

#### Orthologue selection

The function of the candidate genes and their involvement in neurodegeneration was tested in two animal models; *C. elegans* and *Drosophila*. The DRSC Integrated Ortholog Prediction Tool (DIOPT) [104] was used to identify the conserved homologs of human genes in the nematode or fly genomes. Orthologues were defined based on a minimum unweighted DIOPT score of 2, such that two independent bioinformatics algorithms were in agreement concerning the orthologue pairing. In cases where multiple genes were identified as potential orthologues for a given human gene, we carried forward all candidates with DIOPT scores greater than 3.

#### Fly stocks and husbandry

The human α-synuclein transgenic flies with codon-optimization for *Drosophila* (*UAS-α-synuclein* line #7), were recently described [48] and are available from the Bloomington Stock Center (Bloomington, IN, USA). RNAi transgenic lines were obtained from the Vienna *Drosophila* RNAi Centre (Vienna, Austria) or from Bloomington for the Harvard Transgenic RNAi Project. All RNAi lines used for this study are detailed in Additional file 1: Table S8. The GAL4-UAS system [105] was used for ectopic co-expression of both the α-synuclein and RNAi transgene. The *Rh1-Gal4* driver line (second-chromosome insertion) has been previously described [48, 106]. For screening, individual RNAi (IR) lines or Canton S (as a control) were crossed to animals of the genotype: *Rh1-Gal4/CyO*; *UAS-Syn/TM6B*. All crosses were established at 18 °C and F1 experimental animals (*Rh1-Gal4 / UAS-IR*; *UAS-Syn / +* or *Rh1-Gal4 / +*; *UAS-Syn / UAS-IR*) were shifted to 25 °C within 24 h of eclosion and aged 15 days. To examine for potential α-synuclein independent retinal degeneration, each *UAS-IR* transgenic line was separately crossed to *Rh1-Gal4*, using identical conditions. Based on the results of the primary RNAi screen, we also obtained from Bloomington available mutant alleles for the fly orthologues of *PTPRH*: *Ptp10D* and *Ptp4E.* The following additional stocks were used: (1) *w, Ptp4E* ^*1*^; (2) *w, Ptp10D* ^*1*^; (3) *yw, Ptp4E* ^*1*^ *, Ptp10D* ^*1*^ */ FM7C*. All experimental results were quantified and photographed in female animals.

#### Characterization of retinal degeneration in Drosophila

For optical neutralization (also known as the pseudopupil preparation), fly heads of 15-day-old animals were immersed in mineral oil and transilluminated using a 40× objective on a Leica (Wetzlar, Germany) DM6000B light microscope. Eyes from at least four animals were examined per genotype (at least eight retinae). All candidate modifier lines and controls were scored blinded by three independent examiners. The penetrance of degeneration caused by each RNAi line was calculated by dividing the number of abnormal retinae, showing evidence of either reduced rhabodomere numbers or altered refraction of light indicative of vacuolar changes, by the total number of retinae examined. For identification of genetic enhancers, we required two independent RNAi lines targeting non-overlapping sequences with 50% or greater degenerate retinaes observed using the pseudopupil assay. Following our initial screen of two RNAi lines targeting each of 18 fly gene homologs, additional RNAi lines and mutant strains were evaluated, where possible, for the most promising candidates. For each enhancer gene, the strongest RNAi line was independently re-tested for consistency using the pseudopupil assay and retinal histologic sections were also performed for further confirmation. To examine for potential α-synuclein-independent retinal degeneration, the strongest RNAi modifier for each gene was separately crossed to *Rh1-Gal4* and histologic sections were examined for 15-day-old animals. For histology, fly heads from 15-day-old animals were fixed in 8% glutaraldehyde and embedded in paraffin. Tangential (3 μm) retinal sections were cut using a Leica Microtome (RM2245) and stained with hematoxylin and eosin. Retinae from at least three animals were examined and quantified per genotype. Enhancement of α-synuclein-induced retinal degeneration was quantified based on the severity of retinal vacuolar changes seen in stained histologic sections. We examined representative photographs taken with a 40× objective from well-oriented, intact tangential sections at a depth in which the retina achieves maximal diameter. Using ImageJ software [107], we recorded the area occupied by all vacuoles with a diameter greater than 4 μm and divided by the total retinal area to compute a percentage. Statistical comparisons were implemented using a two-tailed student’s t-test. α-synuclein expression levels were determined by immunoblot (clone 42, 1:1000, BD Transduction Laboratories, San Diego, CA, USA).

#### C. elegans media and strains

All strains were maintained as described previously [108]. For this study, the worm strains N2 (wildtype), CF512 (*fer-15(b26)II; fem-1(hc17)III*), and OW40 (zgIs15[P(unc-54)::α-synuclein::YFP]IV) were used. Strains were grown at 20 °C on Nematode Growth medium (NGM) seeded with *Escherichia coli* stain OP50. For each orthologue, one RNAi clone was selected to target the corresponding gene.

#### Phenotype assays for basal phenotypes in C. elegans

The systematic RNAi screen was carried out as described [109]. RNAi clones targeting the genes of interest (9/27; Additional file 1: Table S3) were obtained from the Vidal cDNA RNAi library or the Ahringer RNAi library. Bacteria expressing the empty vector L4440 were used as negative control. For the survival assay, we employed a sterile strain, CF512 (*fer-15(b26); fem-1(hc17*)) [110]. To induce sterility, eggs were collected and kept in M9 medium at 25 °C overnight until they reached L1 arrest. Approximately 25 L1 worms were added to plates seeded with RNAi clones of interest and empty vector control and allowed to develop to adults at 25 °C. At day 9 of adulthood at 25 °C, when approximately half of the worms grown on control plates were dead, the survival of worms on RNAi plates was determined.

The offspring and developmental phenotypes were tested in a single assay. N2 worms were grown at 20 °C until L4 stage on OP50 bacteria and then transferred to plates seeded with RNAi clones of interest and empty vector control. At day 2 of adulthood, ten worms were put onto a new plate seeded with the same RNAi clone for 1 h to produce progeny. The plates containing the progeny were kept at 20 °C until the F1 generation of the control worms reached L4 stage. The number and developmental phenotypes of the offspring were scored at the last time point using a dissecting microscope. A one-sided student’s t-test was used to determine the significant changes compared to controls. All counting was done in a blind fashion in which the identity of the samples was concealed and each experiment was performed in three biological replicates.

#### Motility assay for α-synuclein toxicity model in C. elegans

Animals were age-synchronized by hypochlorite treatment, hatched overnight in M9 buffer, and subsequently cultured on NGM containing isopropylthio-β-D-galactoside (IPTG, 15 mg/L) and 50 μg/mL ampicillin (plates for RNAi treatment). Plates were seeded with RNAi bacteria. Prior to the experiment, the plates were kept at room temperature for two days to allow the production of dsRNA by the bacteria. On day 1 of adulthood (one day after larval stage L4), animals were transferred to RNAi plates containing 5-fluoro-2’deoxy-uridine (FUDR) to prevent the offspring from growing. RNAi clones targeting C54D2.4 (*ARSB*), T08G11.1 (*VPS13C*), and F44G4.8 (*PTPRH*) were used from the Ahringer *C. elegans* RNAi library. All clones were verified by sequencing. RNAi clones for the *C. elegans* orthologue F21F3.7 (*TMEM134*) was not available.

Animals were scored at day 4 and day 8 of adulthood. Animals were placed in a drop of M9 and allowed to adjust for 30 s, after which the number of body bends was counted for another 30 s. Fifteen animals were scored per condition. Relative body bends were calculated by normalizing to control values. Error bars are showing the standard error of mean. Assays were repeated in three independent experiments and the relative body bends of one representative experiment is shown.

## Additional files

Additional file 1: Includes all 12 additional tables with every table on a separate spreadsheet. The file format is an Excel spreadsheet. (XLSX 183 kb)
Additional file 2: Includes all eight additional figures with every figure on a separate slide with the exception of Additional file 1: Figure S1 (divided over two slides). The file formats are PowerPoint (.pptx) and pdf. (PDF 2504 kb)

## Abbreviations

ESP6500: Exome Sequencing Project v. 6500
ExAC: Exome Aggregation Consortium
FBS: Fetal bovine serum
GCN: Gene co-expression network
GO: Gene ontology
GRIP: Genetic Research in Isolated Population
GTEx: The Genotype-Tissue Expression
GWAS: Genome-wide association study
indels: Insertions/deletions
IPDGC: International Parkinson’s Disease Genomics Consortium
IR: Interfering RNA
LoF: Loss of function
MAF: Minor allele frequency
MPS VI: Mucopolysaccharidosis type VI
NGM: Nematode Growth medium
PD: Parkinson’s disease
PPMI: Parkinson Progression Markers Initiative
RNAi: RNA-interference
SNVs: Single nucleotide variants
SSMD: Strictly standardized median difference
UKBEC: United Kingdom Brain Expression Consortium
WES: Whole-exome sequencing
WGCNA: Weighted gene co-expression network analysis
YOPD: Young-onset Parkinson’s disease

## References

[1] Zimprich A, Benet-Pages A, Struhal W, Graf E, Eck SH, Offman MN (2011). A mutation in VPS35, encoding a subunit of the retromer complex, causes late-onset Parkinson disease. Am J Hum Genet..

[2] Vilarino-Guell C, Wider C, Ross OA, Dachsel JC, Kachergus JM, Lincoln SJ (2011). VPS35 mutations in Parkinson disease. Am J Hum Genet..

[3] Funayama M, Ohe K, Amo T, Furuya N, Yamaguchi J, Saiki S (2015). CHCHD2 mutations in autosomal dominant late-onset Parkinson’s disease: a genome-wide linkage and sequencing study. Lancet Neurol..

[4] Farlow JL, Robak LA, Hetrick K, Bowling K, Boerwinkle E, Coban-Akdemir ZH (2016). Whole-exome sequencing in familial Parkinson disease. JAMA Neurol..

[5] Shulman JM, De Jager PL, Feany MB (2011). Parkinson’s disease: genetics and pathogenesis. Annu Rev Pathol..

[6] Trinh J, Farrer M (2013). Advances in the genetics of Parkinson disease. Nat Rev Neurol..

[7] Nalls MA, Pankratz N, Lill CM, Do CB, Hernandez DG, Saad M (2014). Large-scale meta-analysis of genome-wide association data identifies six new risk loci for Parkinson’s disease. Nat Genet..

[8] Hamza TH, Payami H (2010). The heritability of risk and age at onset of Parkinson's disease after accounting for known genetic risk factors. J Hum Genet..

[9] Keller MF, Saad M, Bras J, Bettella F, Nicolaou N, Simon-Sanchez J (2012). Using genome-wide complex trait analysis to quantify ‘missing heritability’ in Parkinson’s disease. Hum Mol Genet..

[10] Jonsson T, Atwal JK, Steinberg S, Snaedal J, Jonsson PV, Bjornsson S (2012). A mutation in APP protects against Alzheimer’s disease and age-related cognitive decline. Nature..

[11] Jonsson T, Stefansson H, Steinberg S, Jonsdottir I, Jonsson PV, Snaedal J (2013). Variant of TREM2 associated with the risk of Alzheimer’s disease. N Engl J Med..

[12] Guerreiro R, Wojtas A, Bras J, Carrasquillo M, Rogaeva E, Majounie E (2013). TREM2 variants in Alzheimer’s disease. N Engl J Med..

[13] Smith BN, Ticozzi N, Fallini C, Gkazi AS, Topp S, Kenna KP (2014). Exome-wide rare variant analysis identifies TUBA4A mutations associated with familial ALS. Neuron..

[14] Cirulli ET, Lasseigne BN, Petrovski S, Sapp PC, Dion PA, Leblond CS (2015). Exome sequencing in amyotrophic lateral sclerosis identifies risk genes and pathways. Science..

[15] Moutsianas L, Agarwala V, Fuchsberger C, Flannick J, Rivas MA, Gaulton KJ (2015). The power of gene-based rare variant methods to detect disease-associated variation and test hypotheses about complex disease. PLoS Genet..

[16] Sulem P, Helgason H, Oddson A, Stefansson H, Gudjonsson SA, Zink F (2015). Identification of a large set of rare complete human knockouts. Nat Genet..

[17] Kitada T, Asakawa S, Hattori N, Matsumine H, Yamamura Y, Minoshima S (1998). Mutations in the parkin gene cause autosomal recessive juvenile parkinsonism. Nature..

[18] Bonifati V, Rizzu P, Squitieri F, Krieger E, Vanacore N, van Swieten JC (2003). DJ-1(PARK7), a novel gene for autosomal recessive, early onset parkinsonism. Neurol Sci..

[19] Valente EM, Abou-Sleiman PM, Caputo V, Muqit MM, Harvey K, Gispert S (2004). Hereditary early-onset Parkinson’s disease caused by mutations in PINK1. Science..

[20] Pringsheim T, Jette N, Frolkis A, Steeves TD (2014). The prevalence of Parkinson’s disease: a systematic review and meta-analysis. Mov Disord..

[21] Lesage S, Drouet V, Majounie E, Deramecourt V, Jacoupy M, Nicolas A (2016). Loss of VPS13C function in autosomal-recessive Parkinsonism causes mitochondrial dysfunction and increases PINK1/Parkin-dependent mitophagy. Am J Hum Genet..

[22] Ghahramani Seno MM, Kwan BY, Lee-Ng KK, Moessner R, Lionel AC, Marshall CR (2011). Human PTCHD3 nulls: rare copy number and sequence variants suggest a non-essential gene. BMC Med Genet..

[23] Newsome TP, Schmidt S, Dietzl G, Keleman K, Asling B, Debant A (2000). Trio combines with dock to regulate Pak activity during photoreceptor axon pathfinding in Drosophila. Cell..

[24] Neumuller RA, Richter C, Fischer A, Novatchkova M, Neumuller KG, Knoblich JA (2011). Genome-wide analysis of self-renewal in Drosophila neural stem cells by transgenic RNAi. Cell Stem Cell..

[25] Ma XM, Kiraly DD, Gaier ED, Wang Y, Kim EJ, Levine ES (2008). Kalirin-7 is required for synaptic structure and function. J Neurosci..

[26] Mandela P, Yankova M, Conti LH, Ma XM, Grady J, Eipper BA (2012). Kalrn plays key roles within and outside of the nervous system. BMC Neurosci..

[27] Greenamyre JT, Hastings TG (2004). Biomedicine. Parkinson’s--divergent causes, convergent mechanisms. Science.

[28] Haelterman NA, Yoon WH, Sandoval H, Jaiswal M, Shulman JM, Bellen HJ (2014). A mitocentric view of Parkinson’s disease. Annu Rev Neurosci..

[29] Pickrell AM, Youle RJ (2015). The roles of PINK1, parkin, and mitochondrial fidelity in Parkinson’s disease. Neuron..

[30] Cookson MR (2012). Parkinsonism due to mutations in PINK1, parkin, and DJ-1 and oxidative stress and mitochondrial pathways. Cold Spring Harb Perspect Med..

[31] Narendra D, Tanaka A, Suen DF, Youle RJ (2008). Parkin is recruited selectively to impaired mitochondria and promotes their autophagy. J Cell Biol..

[32] Kamp F, Exner N, Lutz AK, Wender N, Hegermann J, Brunner B (2010). Inhibition of mitochondrial fusion by alpha-synuclein is rescued by PINK1, Parkin and DJ-1. EMBO J..

[33] Koopman WJ, Visch HJ, Smeitink JA, Willems PH (2006). Simultaneous quantitative measurement and automated analysis of mitochondrial morphology, mass, potential, and motility in living human skin fibroblasts. Cytometry A..

[34] Chang CR, Blackstone C (2010). Dynamic regulation of mitochondrial fission through modification of the dynamin-related protein Drp1. Ann N Y Acad Sci..

[35] Narendra DP, Jin SM, Tanaka A, Suen DF, Gautier CA, Shen J (2010). PINK1 is selectively stabilized on impaired mitochondria to activate Parkin. PLoS Biol..

[36] Vives-Bauza C, Zhou C, Huang Y, Cui M, de Vries RL, Kim J (2010). PINK1-dependent recruitment of Parkin to mitochondria in mitophagy. Proc Natl Acad Sci U S A..

[37] Geisler S, Holmstrom KM, Skujat D, Fiesel FC, Rothfuss OC, Kahle PJ (2010). PINK1/Parkin-mediated mitophagy is dependent on VDAC1 and p62/SQSTM1. Nat Cell Biol..

[38] Vincow ES, Merrihew G, Thomas RE, Shulman NJ, Beyer RP, MacCoss MJ (2013). The PINK1-Parkin pathway promotes both mitophagy and selective respiratory chain turnover in vivo. Proc Natl Acad Sci U S A..

[39] Feany MB, Bender WW (2000). A Drosophila model of Parkinson’s disease. Nature..

[40] Auluck PK, Chan HY, Trojanowski JQ, Lee VM, Bonini NM (2002). Chaperone suppression of alpha-synuclein toxicity in a Drosophila model for Parkinson’s disease. Science..

[41] MacLeod DA, Rhinn H, Kuwahara T, Zolin A, Di Paolo G, McCabe BD (2013). RAB7L1 interacts with LRRK2 to modify intraneuronal protein sorting and Parkinson’s disease risk. Neuron..

[42] Chen L, Feany MB (2005). Alpha-synuclein phosphorylation controls neurotoxicity and inclusion formation in a Drosophila model of Parkinson disease. Nat Neurosci..

[43] Cullen V, Lindfors M, Ng J, Paetau A, Swinton E, Kolodziej P (2009). Cathepsin D expression level affects alpha-synuclein processing, aggregation, and toxicity in vivo. Mol Brain..

[44] Petrucelli L, O’Farrell C, Lockhart PJ, Baptista M, Kehoe K, Vink L (2002). Parkin protects against the toxicity associated with mutant alpha-synuclein: proteasome dysfunction selectively affects catecholaminergic neurons. Neuron..

[45] Yang Y, Nishimura I, Imai Y, Takahashi R, Lu B (2003). Parkin suppresses dopaminergic neuron-selective neurotoxicity induced by Pael-R in Drosophila. Neuron..

[46] Miura E, Hasegawa T, Konno M, Suzuki M, Sugeno N, Fujikake N (2014). VPS35 dysfunction impairs lysosomal degradation of alpha-synuclein and exacerbates neurotoxicity in a Drosophila model of Parkinson’s disease. Neurobiol Dis..

[47] Dhungel N, Eleuteri S, Li LB, Kramer NJ, Chartron JW, Spencer B (2015). Parkinson’s disease genes VPS35 and EIF4G1 interact genetically and converge on alpha-synuclein. Neuron..

[48] Chouhan AK, Guo C, Hsieh Y-C, Ye H, Senturk M, Zuo Z (2016). Uncoupling neuronal death and dysfunction in Drosophila models of neurodegenerative disease. Acta Neuropathol Comm..

[49] van Ham TJ, Holmberg MA, van der Goot AT, Teuling E, Garcia-Arencibia M, Kim HE (2010). Identification of MOAG-4/SERF as a regulator of age-related proteotoxicity. Cell..

[50] Jeon M, Nguyen H, Bahri S, Zinn K (2008). Redundancy and compensation in axon guidance: genetic analysis of the Drosophila Ptp10D/Ptp4E receptor tyrosine phosphatase subfamily. Neural Dev..

[51] Parkinson Progression Marker Initiative. The Parkinson Progression Marker Initiative (PPMI). Prog Neurobiol. 2011;95:629–35 10.1016/j.pneurobio.2011.09.005 PMC9014725 21930184

[52] Pardo LM, MacKay I, Oostra B, van Duijn CM, Aulchenko YS (2005). The effect of genetic drift in a young genetically isolated population. Ann Hum Genet..

[53] Nalls MA, Bras J, Hernandez DG, Keller MF, Majounie E, Renton AE (2015). NeuroX, a fast and efficient genotyping platform for investigation of neurodegenerative diseases. Neurobiol Aging..

[54] Yang J, Benyamin B, McEvoy BP, Gordon S, Henders AK, Nyholt DR (2010). Common SNPs explain a large proportion of the heritability for human height. Nat Genet..

[55] Xu J, Bai J, Zhang X, Lv Y, Gong Y, Liu L (2016). A comprehensive overview of lncRNA annotation resources. Brief Bioinform.

[56] GTEx-Consortium. Human genomics (2015). The Genotype-Tissue Expression (GTEx) pilot analysis: multitissue gene regulation in humans. Science.

[57] Aravind L, Koonin EV (1999). G-patch: a new conserved domain in eukaryotic RNA-processing proteins and type D retroviral polyproteins. Trends Biochem Sci..

[58] Lin ML, Fukukawa C, Park JH, Naito K, Kijima K, Shimo A (2009). Involvement of G-patch domain containing 2 overexpression in breast carcinogenesis. Cancer Sci..

[59] Hu F, Gou L, Liu Q, Zhang W, Luo M, Zhang X (2015). G-patch domain containing 2, a gene highly expressed in testes, inhibits nuclear factor-kappaB and cell proliferation. Mol Med Rep..

[60] Otto GP, Razi M, Morvan J, Stenner F, Tooze SA (2010). A novel syntaxin 6-interacting protein, SHIP164, regulates syntaxin 6-dependent sorting from early endosomes. Traffic..

[61] Wang S, Bellen HJ (2015). The retromer complex in development and disease. Development..

[62] Wang W, Wang X, Fujioka H, Hoppel C, Whone AL, Caldwell MA (2016). Parkinson’s disease-associated mutant VPS35 causes mitochondrial dysfunction by recycling DLP1 complexes. Nat Med..

[63] Song P, Trajkovic K, Tsunemi T, Krainc D (2016). Parkin modulates endosomal organization and function of the endo-lysosomal pathway. J Neurosci..

[64] Chen L, Xie Z, Turkson S, Zhuang X (2015). A53T human alpha-synuclein overexpression in transgenic mice induces pervasive mitochondria macroautophagy defects preceding dopamine neuron degeneration. J Neurosci..

[65] Matozaki T, Suzuki T, Uchida T, Inazawa J, Ariyama T, Matsuda K (1994). Molecular cloning of a human transmembrane-type protein tyrosine phosphatase and its expression in gastrointestinal cancers. J Biol Chem..

[66] Matozaki T, Murata Y, Mori M, Kotani T, Okazawa H, Ohnishi H (2010). Expression, localization, and biological function of the R3 subtype of receptor-type protein tyrosine phosphatases in mammals. Cell Signal..

[67] Takahashi H, Craig AM (2013). Protein tyrosine phosphatases PTPdelta, PTPsigma, and LAR: presynaptic hubs for synapse organization. Trends Neurosci..

[68] Qian M, Pan G, Sun L, Feng C, Xie Z, Tully T (2007). Receptor-like tyrosine phosphatase PTP10D is required for long-term memory in Drosophila. J Neurosci..

[69] Lee HK, Cording A, Vielmetter J, Zinn K (2013). Interactions between a receptor tyrosine phosphatase and a cell surface ligand regulate axon guidance and glial-neuronal communication. Neuron..

[70] Valayannopoulos V, Nicely H, Harmatz P, Turbeville S (2010). Mucopolysaccharidosis VI. Orphanet J Rare Dis..

[71] Tessitore A, Pirozzi M, Auricchio A (2009). Abnormal autophagy, ubiquitination, inflammation and apoptosis are dependent upon lysosomal storage and are useful biomarkers of mucopolysaccharidosis VI. Pathogenetics..

[72] Lieberman AP, Puertollano R, Raben N, Slaugenhaupt S, Walkley SU, Ballabio A (2012). Autophagy in lysosomal storage disorders. Autophagy..

[73] Brooks DA, Gibson GJ, Karageorgos L, Hein LK, Robertson EF, Hopwood JJ (2005). An index case for the attenuated end of the mucopolysaccharidosis type VI clinical spectrum. Mol Genet Metab..

[74] Karageorgos L, Brooks DA, Pollard A, Melville EL, Hein LK, Clements PR (2007). Mutational analysis of 105 mucopolysaccharidosis type VI patients. Hum Mutat..

[75] Sidransky E, Nalls MA, Aasly JO, Aharon-Peretz J, Annesi G, Barbosa ER (2009). Multicenter analysis of glucocerebrosidase mutations in Parkinson’s disease. N Engl J Med..

[76] Sidransky E, Lopez G (2012). The link between the GBA gene and parkinsonism. Lancet Neurol..

[77] Exome Aggregation Consortium (ExAC) C, MA. http://exac.broadinstitute.org [April 2015].

[78] Lek M, Karczewski K, Minikel E, Samocha K, Banks E, Fennell T (2016). Analysis of protein-coding genetic variation in 60,706 humans. Nature..

[79] Dawson TM, Ko HS, Dawson VL (2010). Genetic animal models of Parkinson’s disease. Neuron..

[80] Shulman JM (2015). Drosophila and experimental neurology in the post-genomic era. Exp Neurol..

[81] Li H, Durbin R (2009). Fast and accurate short read alignment with Burrows-Wheeler transform. Bioinformatics..

[82] DePristo MA, Banks E, Poplin R, Garimella KV, Maguire JR, Hartl C (2011). A framework for variation discovery and genotyping using next-generation DNA sequencing data. Nat Genet..

[83] Wang K, Li M, Hakonarson H (2010). ANNOVAR: functional annotation of genetic variants from high-throughput sequencing data. Nucleic Acids Res..

[84] Kircher M, Witten DM, Jain P, O’Roak BJ, Cooper GM (2014). A general framework for estimating the relative pathogenicity of human genetic variants. Nat Genet..

[85] Klein C, Westenberger A (2012). Genetics of Parkinson’s disease. Cold Spring Harb Perspect Med..

[86] Grunewald A, Kasten M, Ziegler A, Klein C (2013). Next-generation phenotyping using the parkin example: time to catch up with genetics. JAMA Neurol..

[87] Exome Variant Server NGESPE, Seattle, WA. http://evs.gs.washington.edu/EVS/ [September 2013 and April 2015].

[88] Abecasis GR, Auton A, Brooks LD, DePristo MA, Durbin RM, Handsaker RE (2012). An integrated map of genetic variation from 1,092 human genomes. Nature..

[89] Li MX, Gui HS, Kwan JS, Bao SY, Sham PC (2012). A comprehensive framework for prioritizing variants in exome sequencing studies of Mendelian diseases. Nucleic Acids Res..

[90] Sherry ST, Ward MH, Kholodov M, Baker J, Phan L, Smigielski EM (2001). dbSNP: the NCBI database of genetic variation. Nucleic Acids Res.

[91] Robinson JT, Thorvaldsdottir H, Winckler W, Guttman M, Lander ES, Getz G (2011). Integrative genomics viewer. Nat Biotechnol..

[92] Ionita-Laza I, Lee S, Makarov V, Buxbaum JD, Lin X (2013). Sequence kernel association tests for the combined effect of rare and common variants. Am J Hum Genet..

[93] Purcell S, Neale B, Todd-Brown K, Thomas L, Ferreira MA, Bender D (2007). PLINK: a tool set for whole-genome association and population-based linkage analyses. Am J Hum Genet..

[94] Simon-Sanchez J, Schulte C, Bras JM, Sharma M, Gibbs JR, Berg D (2009). Genome-wide association study reveals genetic risk underlying Parkinson’s disease. Nat Genet..

[95] International Parkinson’s Disease Genomics Consortium, Wellcome Trust Case Control Consortium (2011). A two-stage meta-analysis identifies several new loci for Parkinson’s disease. PLoS Genet.

[96] Pruim RJ, Welch RP, Sanna S, Teslovich TM, Chines PS, Gliedt TP (2010). LocusZoom: regional visualization of genome-wide association scan results. Bioinformatics..

[97] Langfelder P, Horvath S (2008). WGCNA: an R package for weighted correlation network analysis. BMC Bioinformatics..

[98] Forabosco P, Ramasamy A, Trabzuni D, Walker R, Smith C, Bras J (2013). Insights into TREM2 biology by network analysis of human brain gene expression data. Neurobiol Aging..

[99] Reimand J, Kull M, Peterson H, Hansen J, Vilo J (2007). g:Profiler--a web-based toolset for functional profiling of gene lists from large-scale experiments. Nucleic Acids Res.

[100] Langfelder P, Luo R, Oldham MC, Horvath S (2011). Is my network module preserved and reproducible?. PLoS Comput Biol..

[101] Shannon P, Markiel A, Ozier O, Baliga NS, Wang JT, Ramage D (2003). Cytoscape: a software environment for integrated models of biomolecular interaction networks. Genome Res..

[102] Zhang XD (2011). Illustration of SSMD, z score, SSMD*, z* score, and t statistic for hit selection in RNAi high-throughput screens. J Biomol Screen..

[103] Zhang JH, Chung TD, Oldenburg KR (1999). A simple statistical parameter for use in evaluation and validation of high throughput screening assays. J Biomol Screen..

[104] Hu Y, Flockhart I, Vinayagam A, Bergwitz C, Berger B, Perrimon N (2011). An integrative approach to ortholog prediction for disease-focused and other functional studies. BMC Bioinformatics..

[105] Brand AH, Perrimon N (1993). Targeted gene expression as a means of altering cell fates and generating dominant phenotypes. Development..

[106] Xiong B, Bayat V, Jaiswal M, Zhang K, Sandoval H, Charng WL (2012). Crag is a GEF for Rab11 required for rhodopsin trafficking and maintenance of adult photoreceptor cells. PLoS Biol..

[107] Schneider CA, Rasband WS, Eliceiri KW (2012). NIH Image to ImageJ: 25 years of image analysis. Nat Methods..

[108] Brenner S (1974). The genetics of Caenorhabditis elegans. Genetics..

[109] Hansen M, Hsu AL, Dillin A, Kenyon C (2005). New genes tied to endocrine, metabolic, and dietary regulation of lifespan from a Caenorhabditis elegans genomic RNAi screen. PLoS Genet..

[110] Garigan D, Hsu AL, Fraser AG, Kamath RS, Ahringer J, Kenyon C (2002). Genetic analysis of tissue aging in Caenorhabditis elegans: a role for heat-shock factor and bacterial proliferation. Genetics..

