# Supplementary material for: Discovery and functional prioritization of Parkinson’s disease candidate genes from large-scale whole exome sequencing
Source: Genome Biol. 2017 Jan 30;18:22. doi: 10.1186/s13059-017-1147-9 (PMC5282828; doi:10.1186/s13059-017-1147-9)
Supplement: Additional file 2: — Includes all eight additional figures with every figure on a separate slide with the exception of Additional file 1: Figure S1 (divided over two slides). The file formats are PowerPoint (.pptx) and pdf. (PDF 2504 kb) [file 13059_2017_1147_MOESM2_ESM.pdf]

Figure S1 (A-F)

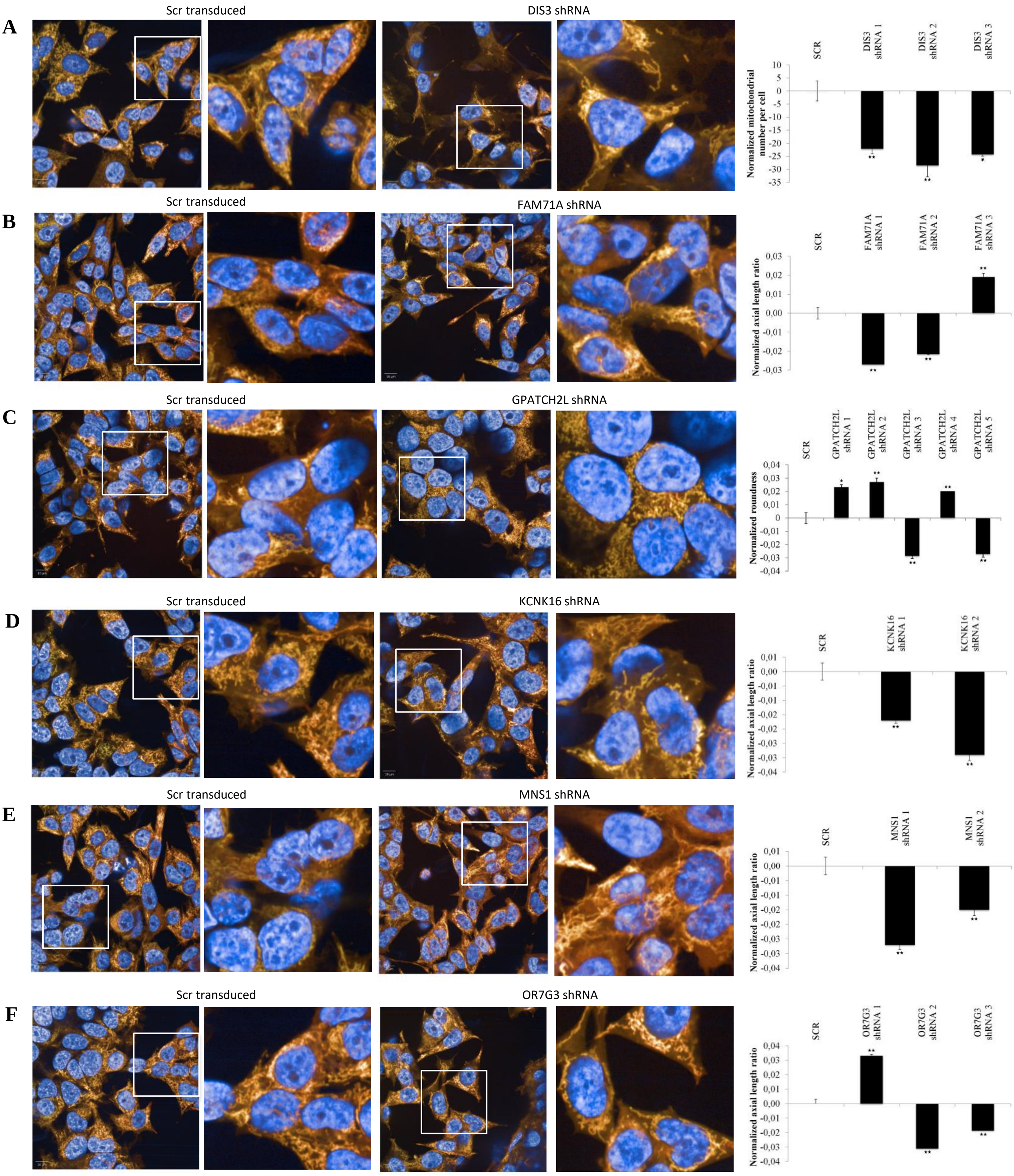
A
Scr transduced
DIS3 shRNA
SCR
DIS3 shRNA 1
DIS3 shRNA 2
DIS3 shRNA 3
Normalized mitochondrial number per cell
B
Scr transduced
FAM71A shRNA
SCR
FAM71A shRNA 1
FAM71A shRNA 2
FAM71A shRNA 3
Normalized axial length ratio
C
Scr transduced
GPATCH2L shRNA
SCR
GPATCH2L shRNA 1
GPATCH2L shRNA 2
GPATCH2L shRNA 3
GPATCH2L shRNA 4
GPATCH2L shRNA 5
Normalized roundness
D
Scr transduced
KCNK16 shRNA
SCR
KCNK16 shRNA 1
KCNK16 shRNA 2
Normalized axial length ratio
E
Scr transduced
MNS1 shRNA
SCR
MNS1 shRNA 1
MNS1 shRNA 2
Normalized axial length ratio
F
Scr transduced
OR7G3 shRNA
SCR
OR7G3 shRNA 1
OR7G3 shRNA 2
OR7G3 shRNA 3
Normalized axial length ratio

Figure S1 (G-L)

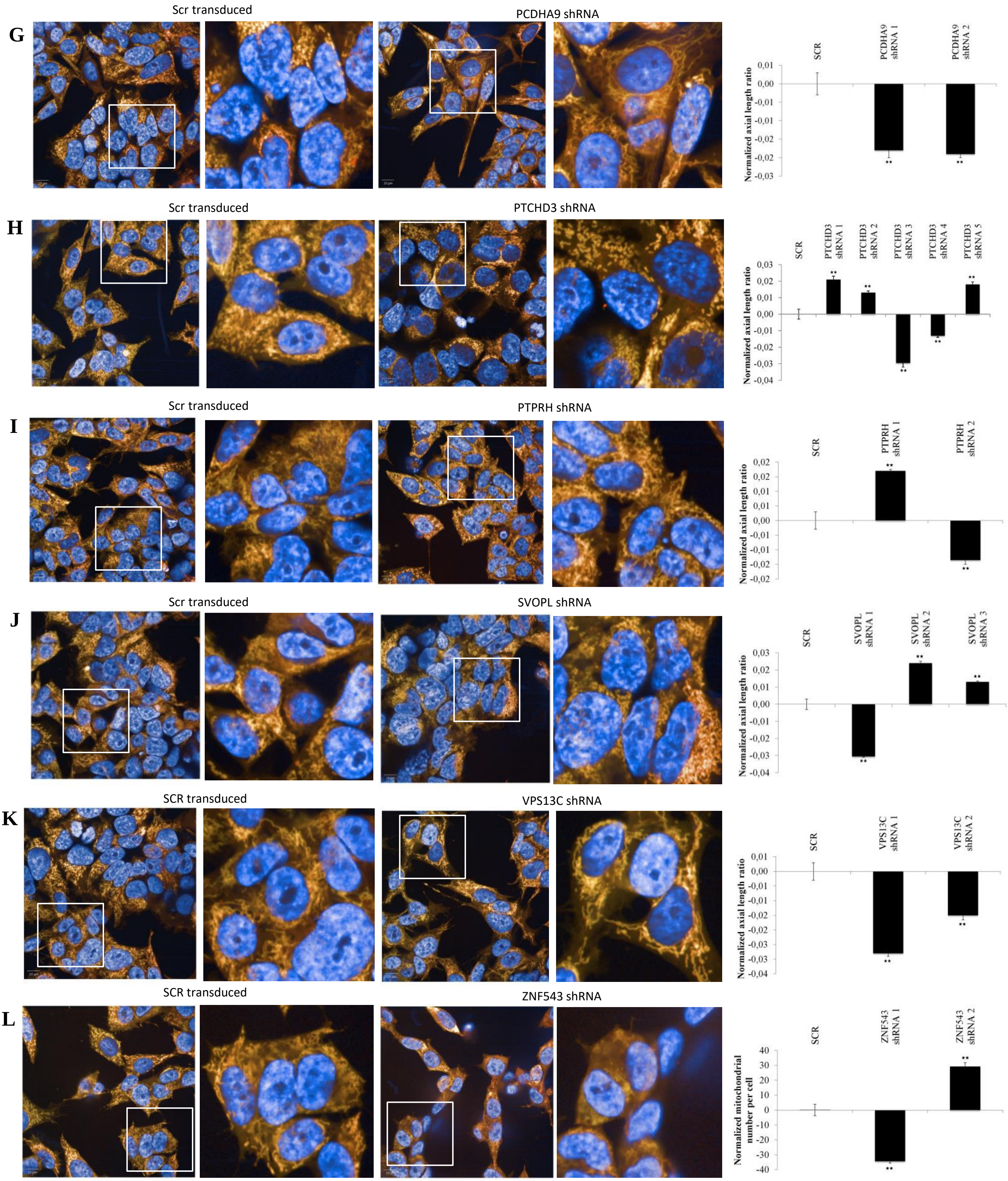
G
Scr transduced
PCDHA9 shRNA
SCR
PCDHA9 shRNA 1
PCDHA9 shRNA 2
Normalized axial length ratio
H
Scr transduced
PTCHD3 shRNA
SCR
PTCHD3 shRNA 1
PTCHD3 shRNA 2
PTCHD3 shRNA 3
PTCHD3 shRNA 4
PTCHD3 shRNA 5
Normalized axial length ratio
I
Scr transduced
PTPRH shRNA
SCR
PTPRH shRNA 1
PTPRH shRNA 2
Normalized axial length ratio
J
Scr transduced
SVOPL shRNA
SCR
SVOPL shRNA 1
SVOPL shRNA 2
SVOPL shRNA 3
Normalized axial length ratio
K
SCR transduced
VPS13C shRNA
SCR
VPS13C shRNA 1
VPS13C shRNA 2
Normalized axial length ratio
L
SCR transduced
ZNF543 shRNA
SCR
ZNF543 shRNA 1
ZNF543 shRNA 2
Normalized mitochondrial number per cell

Figure S1. High Content assay for Mitochondrial Morphology. Images show cells labeled with Hoechst (BLUE; Nuclei) and mitochondria (Mitotracker CMXRos and Mitotracker Deepred, YELLOW). Untreated cells are infected with shRNA with a scrambled sequence (left panel). Cells infected with shRNA targeting the specified gene show a decrease or increase in number/roundness/axial length ratio of mitochondria per cell (right panel). The graph displays normalised numbers of the specified parameters. Data are median values ± median absolute deviation (MAD) of N=6 measurements. *P < 0.05 and ** P < 0.01, Mann-Whitney U test (see methods). All values were normalised to the negative control (infected with shRNA encoding a scrambled sequence). All shRNA clones that meet the cut-off criteria (see methods) are shown. **A)** DIS3 shRNA decreases number of mitochondria per cell. **B)** FAM71A shRNA decreases mitochondrial axial length ratio and roundness. **C)** GPATCH2L shRNA increases mitochondrial roundness. **D)** KCNK16 shRNA decreases mitochondrial axial length ratio and roundness. **E)** MNS1 shRNA decreases mitochondrial axial length ratio. **F)** OR7G3 shRNA decreases mitochondrial axial length ratio and increases mitochondrial roundness and number of mitochondria per cell. **G)** *PCDHA9* shRNA decreases mitochondrial axial length ratio and roundness. **H)** *PTCHD3* shRNA increases mitochondrial axial length ratio and roundness. **I)** *PTPRH* shRNA increases mitochondrial axial length ratio. **J)** *SVOPL* shRNA increases mitochondrial axial length ratio and decreases mitochondrial roundness. **K)** *VPS13C* shRNA decreases axial length ratio. **L)** *ZNF543* shRNA decreases number of mitochondria per cell.

Figure S2

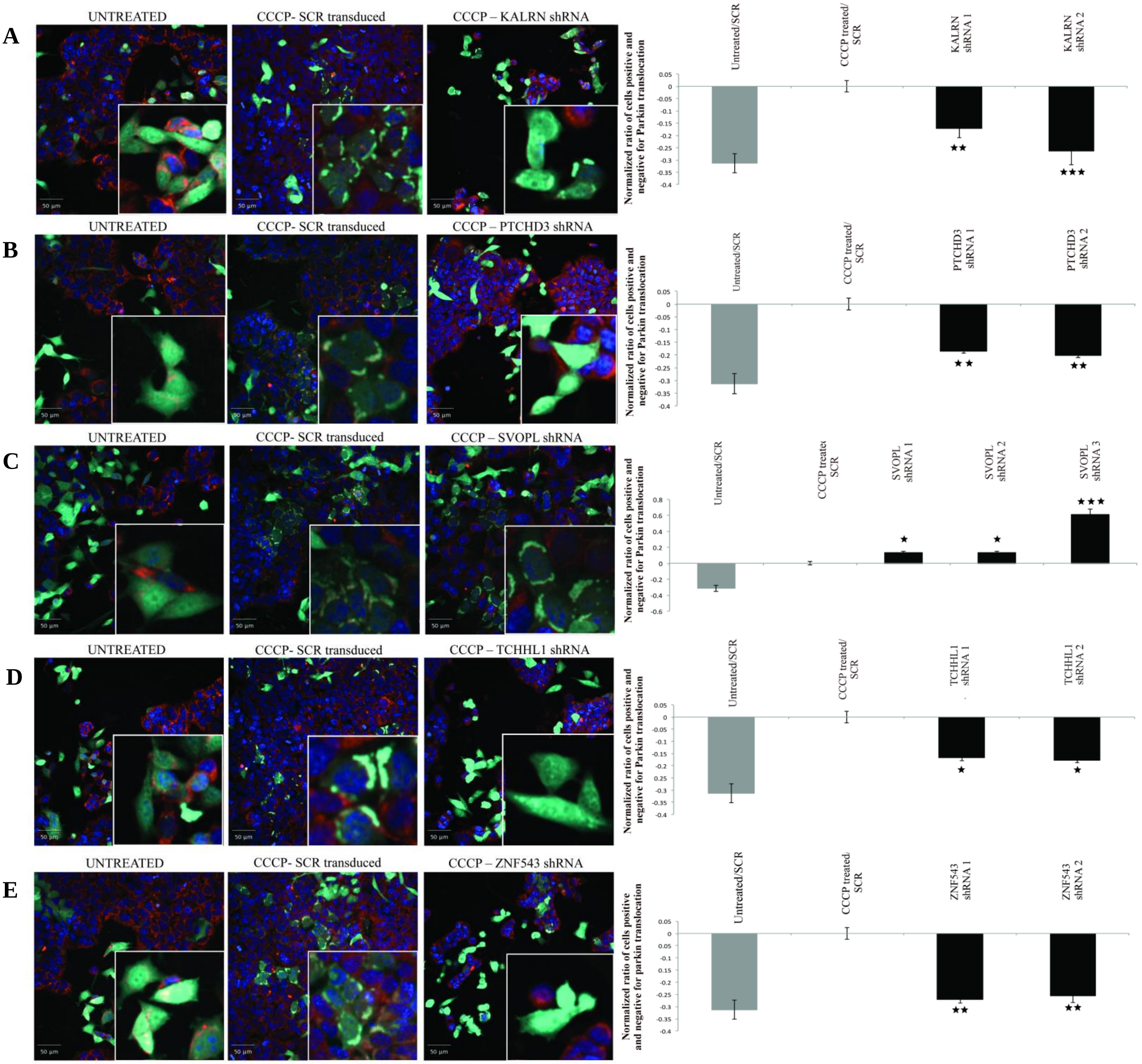

Figure S2. <u>High content assay for Parkin Translocation</u>. Effect of *KALRN* shRNA (A), *PTCHD3* shRNA (B), *SVOPL* shRNA (C), *TCHHL1* shRNA (D) and *ZNF543* shRNA (E). The images display cells labeled for nuclei (BLUE; Hoechst), Parkin-GFP (GREEN), mitochondria (RED, Mitotracker Deepred). Untreated cells infected with shRNA encoding a scrambled sequence show absence of puncta (left panel). Cells infected with a scrambled sequence but treated with CCCP show a significant increase in puncta formation (middle panel). Infection of cells with shRNA targeting *KALRN*, *PTCHD3*, *TCHHL1* and *ZNF543* prevents the accumulation of Parkin on mitochondrial (right panel), while targeting SVOPL promotes this accumulation. The graphs display the normalised ratio of cells positive for translocation and cells negative for Parkin translocation. All values were normalised to the negative control (CCCP treated infected with shRNA encoding a scrambled sequence). Data are median values ± median absolute deviation (MAD) of N=6 measurements. * $P < 0.05$, ** $P < 0.01$ and *** $P < 0.001$, Mann-Whitney U test (see methods).All shRNA clones that meet the cut-off criteria (see methods) are shown.

Figure S3

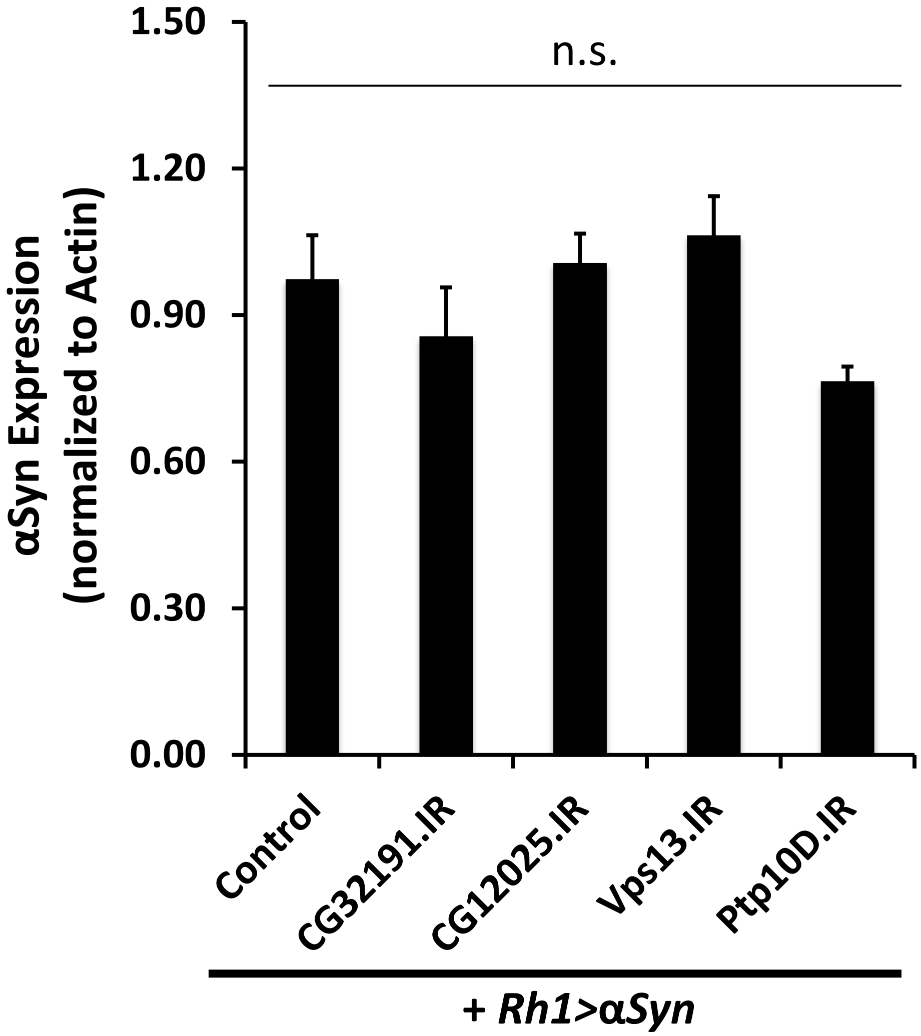

Figure S3. Enhancers of α-synuclein toxicity do not significantly increase α-synuclein protein levels. Western blots were performed on fly heads for *Rh1>α-synuclein* controls and following knockdown of each enhancer (same genotypes detailed in Figure 5 legend). Relative expression of α-synuclein was determined based on normalization to Actin. n.s., non-significant.

Figure S4

A **Day 4**

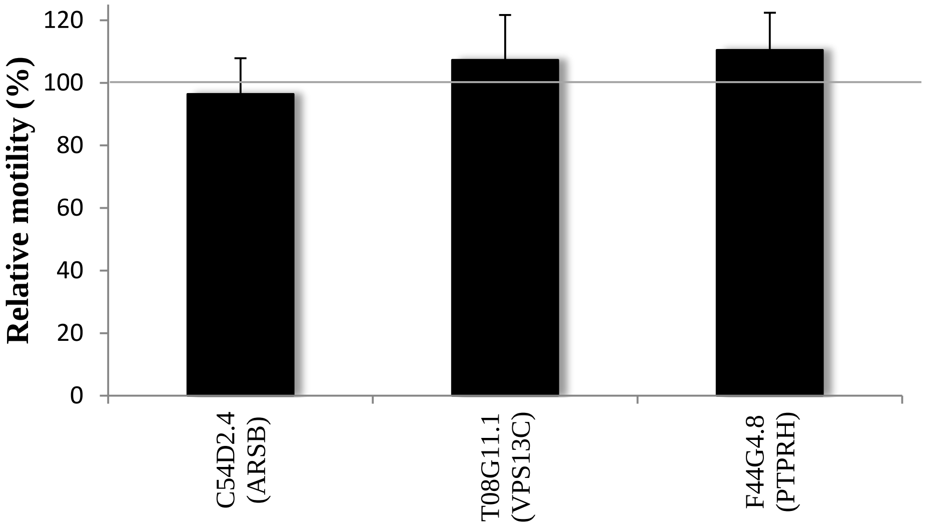

B **Day 8**

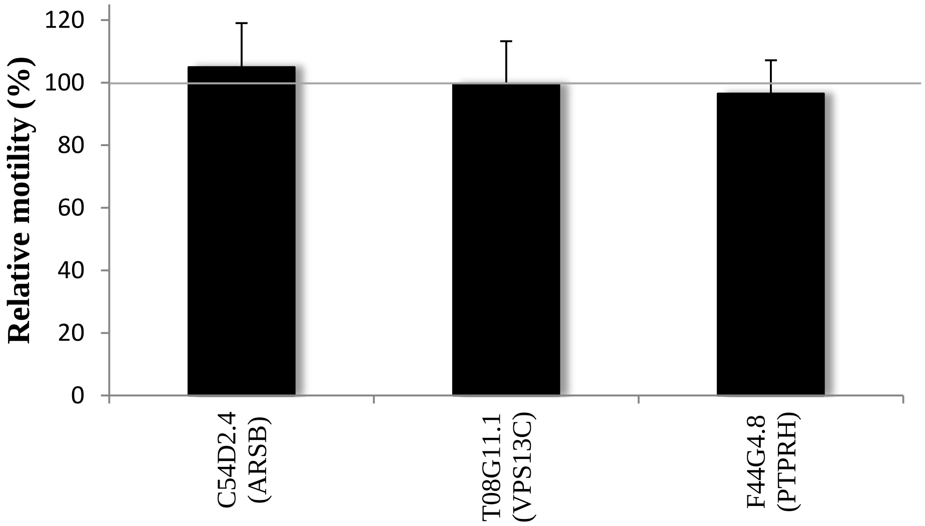

Figure S4. Motility assay in *C. elegans* model overexpressing human α-synculein and YFP in the body wall muscle. Motility relative to the control is displayed for the time points day 4 and day 8. No significant decrease or increase of body movements observed after knockdown by shRNA of the corresponding orthologues.

Figure S5

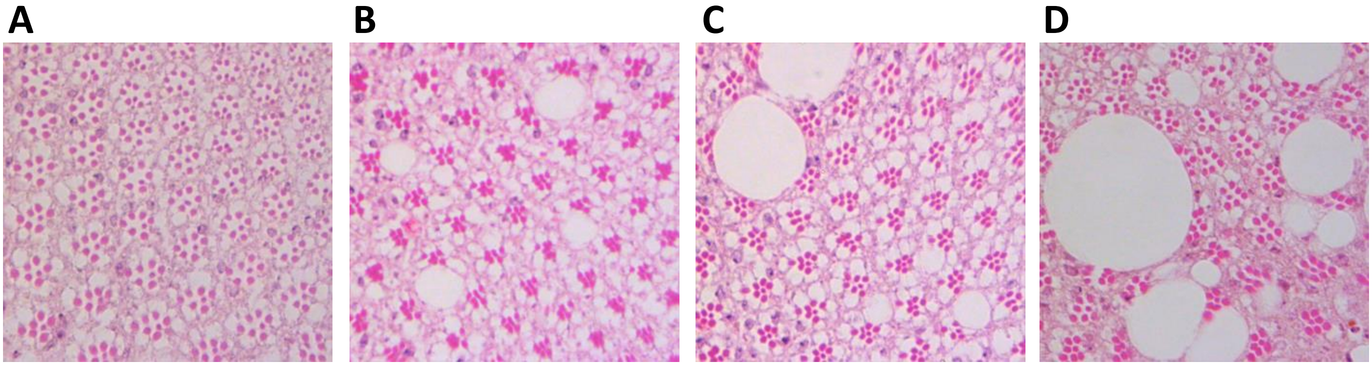

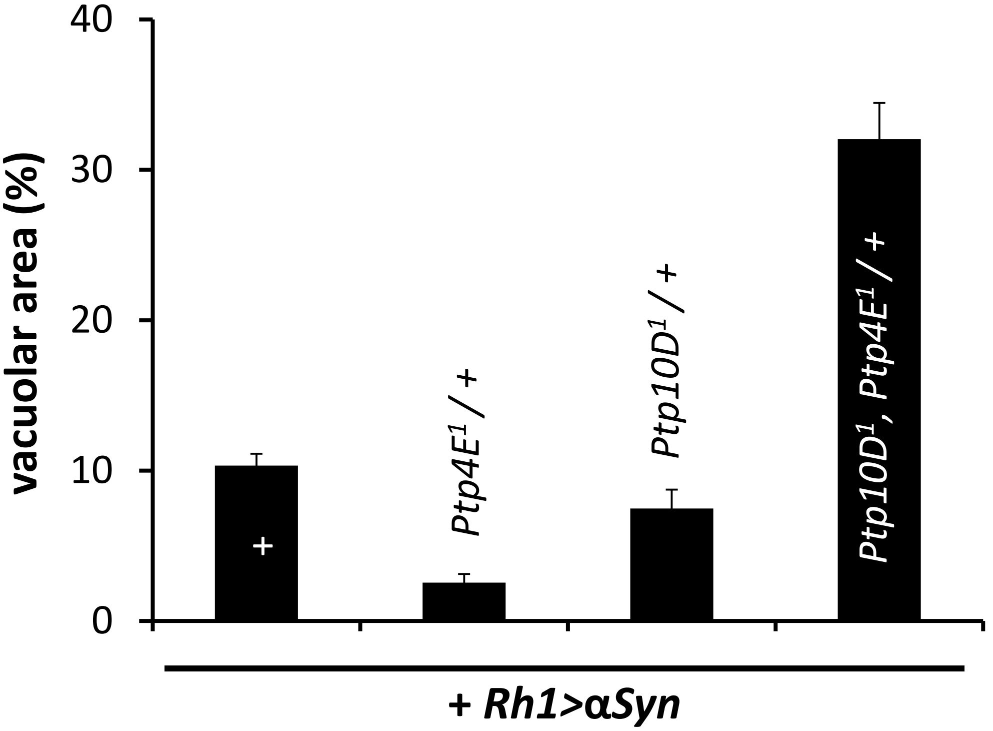

Figure S5. Enhancement of α-synuclein toxicity by *Drosophila* homologs of *PTPRH*. Double-heterozygotes for strong mutant alleles in the paralogous genes, *Ptp10D* and *Ptp4E*, enhance α-synuclein-induced retinal degeneration, whereas neither mutant allele enhances α-synuclein toxicity on its own.

(A) *Ptp10D*^{1}*, Ptp4E*^{1} */ +; Rh1-Gal4 / +*

(B) *Ptp4E*^{1} */ +; Rh1-Gal4 / +; UAS-Syn / +*

(C) *Ptp10D*^{1} */ +; Rh1-Gal4 / +; UAS-Syn / +*

(D) *Ptp10D*^{1}*, Ptp4E*^{1} */ +; Rh1-Gal4/+; UAS-Syn / +*

Figure S6

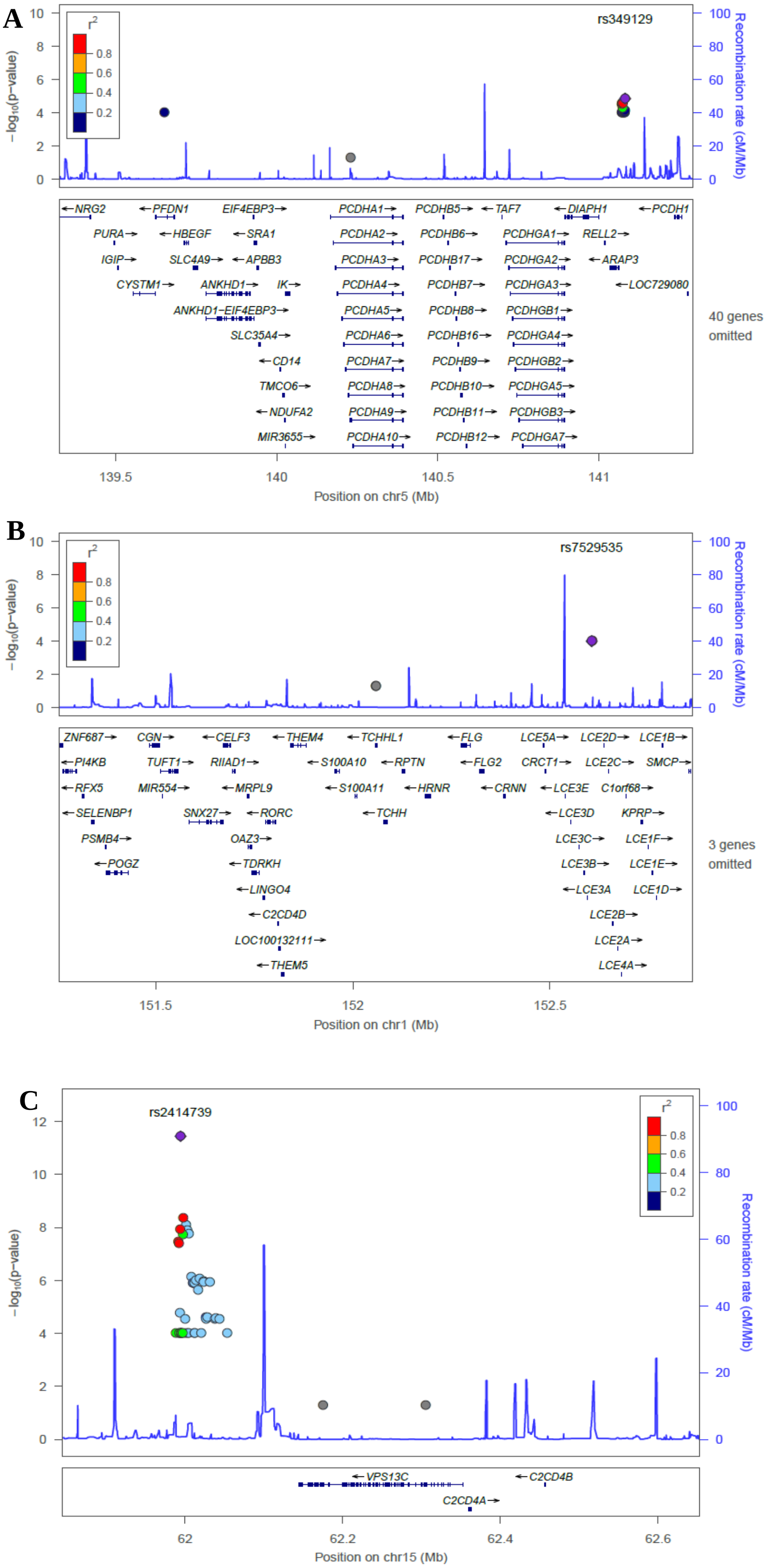

Fig S6. GWAS peak regions around *PCDHA9* (A), *TCHHL1* (B) and *VPS13C* (C) with underlying European LD-structure. The grey dots indicate the location of the LoF variant of the exome data. The colored dots indicate the positions and *p*-values of the GWAS hits.

Figure S7

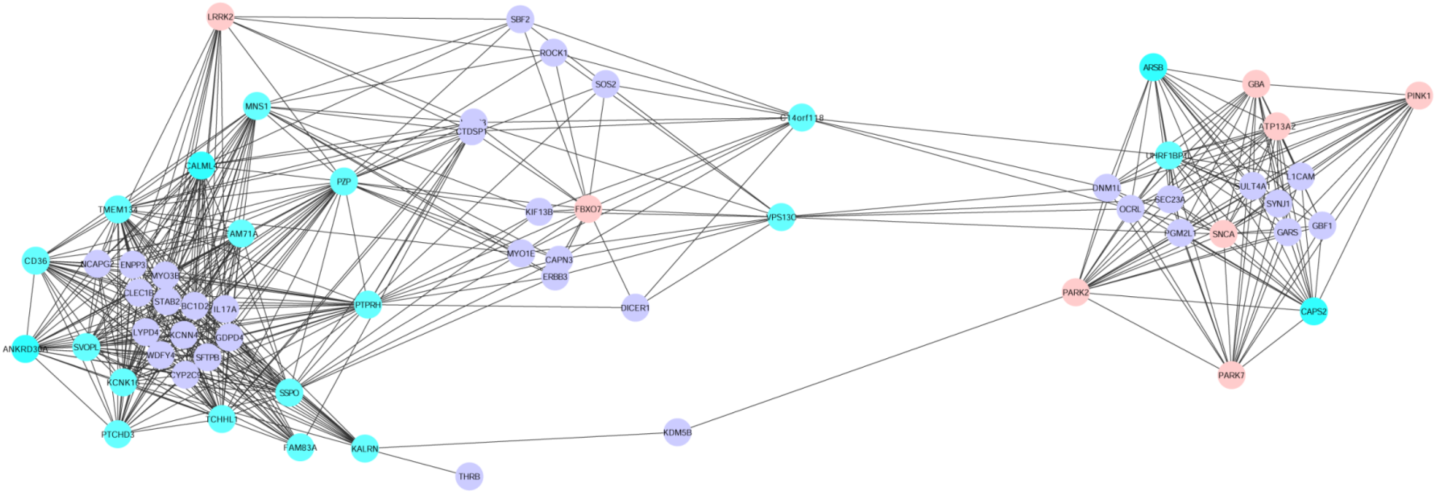

Fig S7. Plot to demonstrate the co-expression of candidate genes with well-established PD genes. We generated a “bottom-up” plot using the 20 candidate genes expressed in substantia nigra and known PD genes as seeds and based on TOM values between genes (edges were filtered for an adjacency cut-off of TOM values < 0.01 to show the most relevant interactions) for the UKBEC GCN. Candidate genes are highlighted in light blue, PD genes in pink and context genes in purple. This plot demonstrate two main subnetworks, the one on the left centered around *FBXO7* and *LRRK2*, the one on the right centered around *ATP13A2*, *GBA*, *PARK2*, *PARK7*, *PINK1* and *SNCA*. The latter shows a tight interaction of *UHRF1BP1L* with the PD genes it clusters with within the yellow module, but also with *PARK2* and *PARK7*.

Figure S8

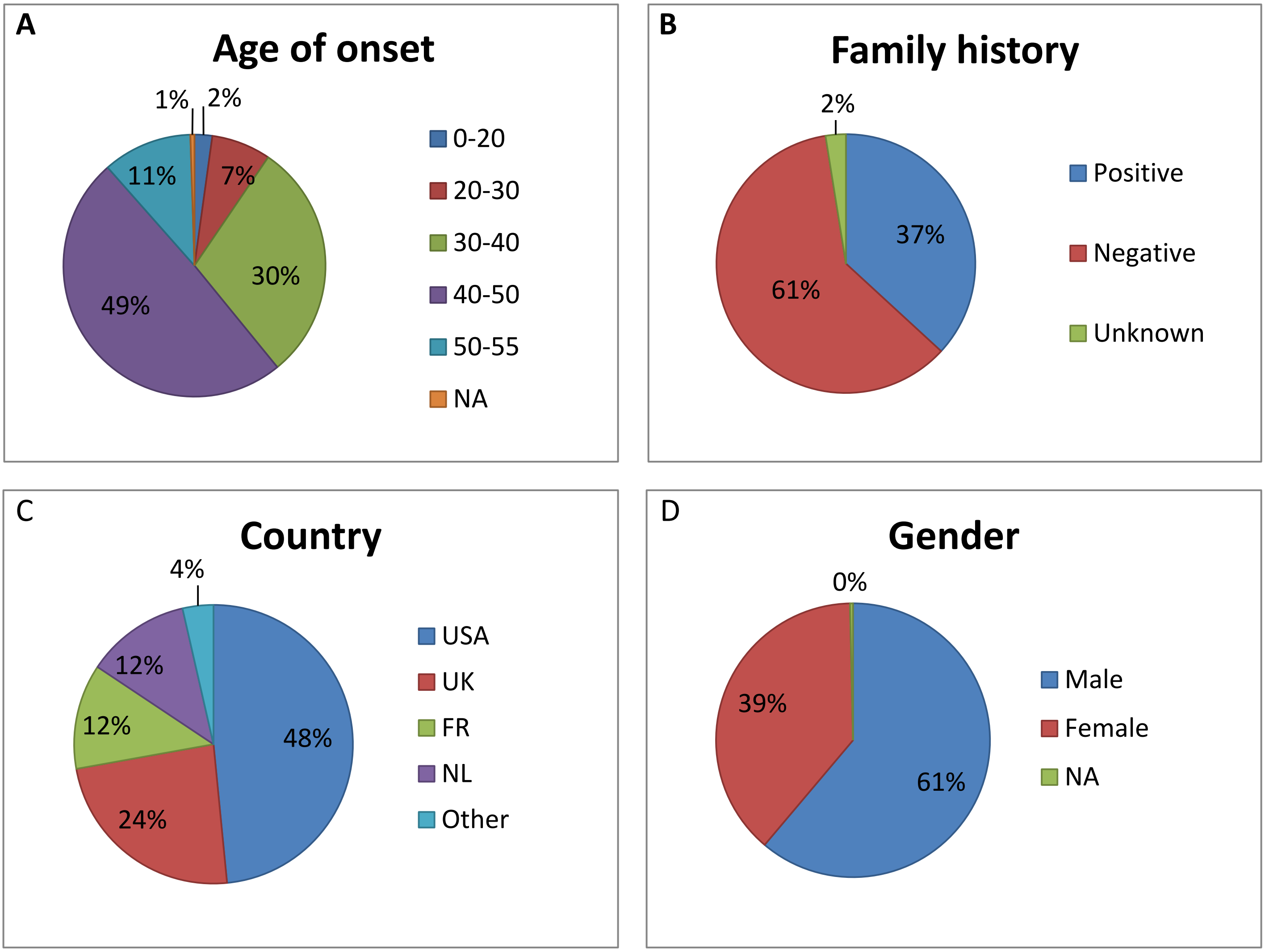

Fig S8. Cohort demographics. A: Age of onset distribution of cases expressed in years (NA identifies PD cases that were labeled as "YOPD" but lacked an age of onset); B. Family history distribution of cases; C. Country of origin distribution of all samples (USA=United States of America; UK=United Kingdom; NL=The Netherlands; FR= France); D. Gender distribution of all samples.
